# Distinct High‐Gamma Signals in Primate Prefrontal Cortex Differentiate Cue Information, Preference Revision, and Expected Reward During Value‐Based Decisions

**DOI:** 10.1111/ejn.70641

**Published:** 2026-08-12

**Authors:** Renée Johnston, Chadwick Boulay, Laurence Hunt, Steven W. Kennerley, Adam J. Sachs

**Affiliations:** ^1^ Ottawa Hospital Research Institute Ottawa Ontario Canada; ^2^ University of Ottawa Brain and Mind Research Institute Ottawa Ontario Canada; ^3^ Department of Experimental Psychology University of Oxford Oxford UK; ^4^ Division of Neurosurgery Ottawa Hospital Research Institute Ottawa Ontario Canada

**Keywords:** decision‐making, local field potentials, support vector machine

## Abstract

Understanding how prefrontal cortex (PFC) signals evolve over time to support decision‐making requires characterizing both the spectral and temporal structure of neural activity. High‐gamma (Hγ) power in local field potentials (LFPs) reflects local population firing, yet its role in differentiating cue‐driven preference revision, reward expectation, and outcome‐related signals during reward‐guided decisions remains unclear. We recorded LFPs from the anterior cingulate cortex (ACC), dorsolateral PFC (DLPFC), and orbitofrontal cortex (OFC) in macaques performing a multicue reward‐based decision task in which up to four sequential cues indicated the expected reward associated with competing targets. Hγ power was extracted in sliding windows aligned to key task events, and support vector machine classifiers were used to decode cue‐driven preference revision, reward expectation, cue value level and position, target choice, and prediction‐error contrasts. Hγ responses robustly differentiated preference‐reversal from confirmation trials, with subject‐specific modulation patterns across PFC subregions. Cue value level and spatial position were reliably decoded across PFC, with stronger differentiation of cue value level in OFC and cue position in DLPFC. Around movement onset, Hγ activity differentiated high versus low expected reward across PFC, with decoding accuracies reaching 85% in ACC. Hγ was also modulated by unexpected reward omission, yielding 78% decoding accuracy from a single OFC channel. These findings demonstrate that Hγ activity across PFC subregions differentiates multiple computationally defined decision variables with distinct temporal profiles and overlapping regional contributions, highlighting Hγ as a robust marker of dynamic reward‐guided decision processes.

## Graphical abstract

High‐gamma activity in primate prefrontal cortex differentiates multiple task‐defined variables during sequential decision‐making, reflecting population‐level dynamics consistent with mixed‐selectivity representations.

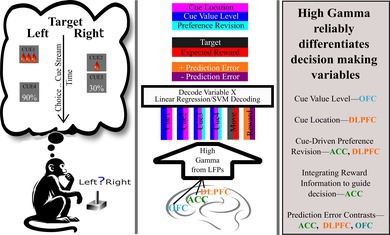

**Abbreviations**
ACC: anterior cingulate cortex
CAR: common average reference
DLPFC: dorsolateral prefrontal cortex
EV: expected value
FDR: false discovery rate
Hγ: high‐gamma
LFP: local field potential
MRI: magnetic resonance imaging
OFC: orbitofrontal cortex
PE: prediction error
PFC: prefrontal cortex
pv: *p* value
SVM: support vector machine

## 1 Introduction

Flexible, value‐based decision‐making is fundamental to adaptive behavior. It requires the brain to integrate sensory inputs, contextual knowledge, and internal goals to guide actions. The prefrontal cortex (PFC) plays a central role in these processes (Miller and Cohen 2001), enabling evaluation of cues, integration of context, and updating of internal models. Although PFC neurons often exhibit mixed selectivity and distributed coding, different PFC subregions have been associated with partially overlapping functional specializations. For example, the anterior cingulate cortex (ACC) has been implicated in predicting action outcomes (Alexander and Brown 2011), signaling prediction errors (Holroyd and Coles 2002), and encoding distributions of reward outcomes in distributional reinforcement learning (Muller et al. 2024); the dorsolateral PFC (DLPFC) contributes to working memory and executive control (Miller et al. 1996; Curtis and D'Esposito 2003), and also plays roles in intention coding (Boulay et al. 2016; Johnston et al. 2021), associative learning (Rouzitalab et al. 2023), spatial representations (Johnston et al. 2023), and attention (Buschman and Miller 2007; Tremblay et al. 2015); and the orbitofrontal cortex (OFC) is involved in value encoding (Padoa‐Schioppa and Assad 2006; Rushworth et al. 2011), reward integration (Wallis 2007), and the construction of cognitive maps and task structure representations that support flexible, goal‐directed behavior (Schuck et al. 2016; Wikenheiser and Schoenbaum 2016).

Despite extensive research, it remains unclear how PFC subregions contribute dynamically to decision‐making and reflect internal variables such as preference revision, expected value (EV), and prediction error. OFC signals can fluctuate between choice options (Rich and Wallis 2016), and PFC subregional activity evolves dynamically during evidence accumulation (Hunt, Malalasekera, de Berker, et al. 2018), suggesting that value signals are flexible and context dependent. While medial and orbitofrontal regions such as ventromedial PFC and OFC encode reward values (Murray and Rudebeck 2018), ACC, lateral premotor, and parietal regions support cue‐based action selection (Cisek and Kalaska 2005; Rushworth et al. 2003; Kang et al. 2025). Many real‐world decisions, however, require integrating perceptual cues to predict potential outcomes.

Electrophysiological studies provide complementary insights into these computations at different scales. Single‐unit recordings reveal selectivity for task variables (Seo and Lee 2007; Hunt et al. 2015) but often lack population‐level temporal dynamics. In contrast, functional MRI provides a broad spatial coverage while maintaining spatial resolution on the order of millimeters, but lacks temporal precision to capture fast cognitive transitions (Logothetis 2008). Local field potentials (LFPs), particularly in the high‐gamma (Hγ, 50–150 Hz) range, offer a powerful intermediate signal: they closely track local ensemble spiking and synaptic activity (Buzsáki et al. 2012), providing millisecond‐level temporal precision and spatial specificity. Hγ also reflects cortical processing and attentional engagement (Crone et al. 1998; Lachaux et al. 2012), making it well‐suited to study dynamic modulation related to cognitive variables.

However, whether and how Hγ power reflects cue‐driven preference revision, when new evidence reverses the currently preferred option, has not been systematically characterized across PFC subregions during reward‐guided behavior. Some studies have reported decision‐related modulation of gamma activity (Pesaran et al. 2008; Hunt et al. 2015), but it remains unclear how Hγ compares to low‐frequency bands such as beta, which are often linked to cognitive stability or motor suppression (Engel and Fries 2010), and how it reflects latent variables such as belief state, expected reward, or prediction error.

Here, we leveraged a rich open‐access dataset (Hunt, Malalasekera, and Kennerley 2018; Hunt, Malalasekera, de Berker, et al. 2018) comprising acute LFP recordings from ACC, DLPFC, and OFC in two macaques performing a sequential, cue‐based reward decision task. Across trials, up to four visual cues conveyed partial reward information, allowing us to define task‐derived decision variables such as cue level and position, cue‐driven preference revision, expected reward, and prediction error. This dataset is well suited to dissociate cue‐driven preference revision from cue level because each cue provides incremental probabilistic evidence about reward.

While prior analyses of these recordings examined neuronal spiking activity and decision‐related computations across PFC (Hunt, Malalasekera, de Berker, et al. 2018), the present study focuses on Hγ LFP dynamics across prefrontal regions. Using time‐resolved spectral and decoding analyses, we test whether task‐defined decision variables are reflected in Hγ activity across PFC subregions. This approach provides insight into how cue‐driven preference revision and reward evaluation unfold dynamically within prefrontal circuits.

## 2 Methods

### 2.1 Subjects and Experimental Setup

Two adult male rhesus macaques ( *Macaca mulatta* ), referred to as Miles and Frank, were implanted with recording chambers positioned over the frontal cortex and performed a sequential cue‐based value decision‐making task previously described by Hunt, Malalasekera, de Berker, et al. (2018). All experimental procedures were approved by the relevant institutional animal care committees and complied with national guidelines for the ethical treatment of animals.

Neural recordings were obtained from the PFC, including the ACC, DLPFC, and OFC. Recording sites were targeted using stereotaxic coordinates based on anatomical landmarks described in the original dataset.

Electrophysiological recordings were obtained using tungsten microelectrodes (FHC Instruments) that were inserted acutely each day through the recording chamber. Signals were amplified, filtered, and digitized using standard electrophysiological acquisition systems, and LFPs were obtained by low‐pass filtering the recorded signals as described in Hunt, Malalasekera, de Berker, et al. (2018).

### 2.2 Recording Sessions and Behavioral Data

Recordings were performed across multiple daily sessions while animals performed the task for liquid reward. Each session consisted of several hundred trials. Animals completed one recording session per day, and the same cue‐reward mapping was maintained across several consecutive days (up to 3 days for Miles and 4 days for Frank).

To examine the temporal evolution of task learning, sessions were grouped relative to the introduction of a new cue‐reward association set. Within each session, cues were sampled from this set in random combinations across trials, resulting in different stimulus sequences across trials and across days. Day 0 corresponded to the first exposure to a new cue set, followed by Days 1–4, during which animals learned and consolidated the cue‐reward mappings.

**FIGURE 1:**
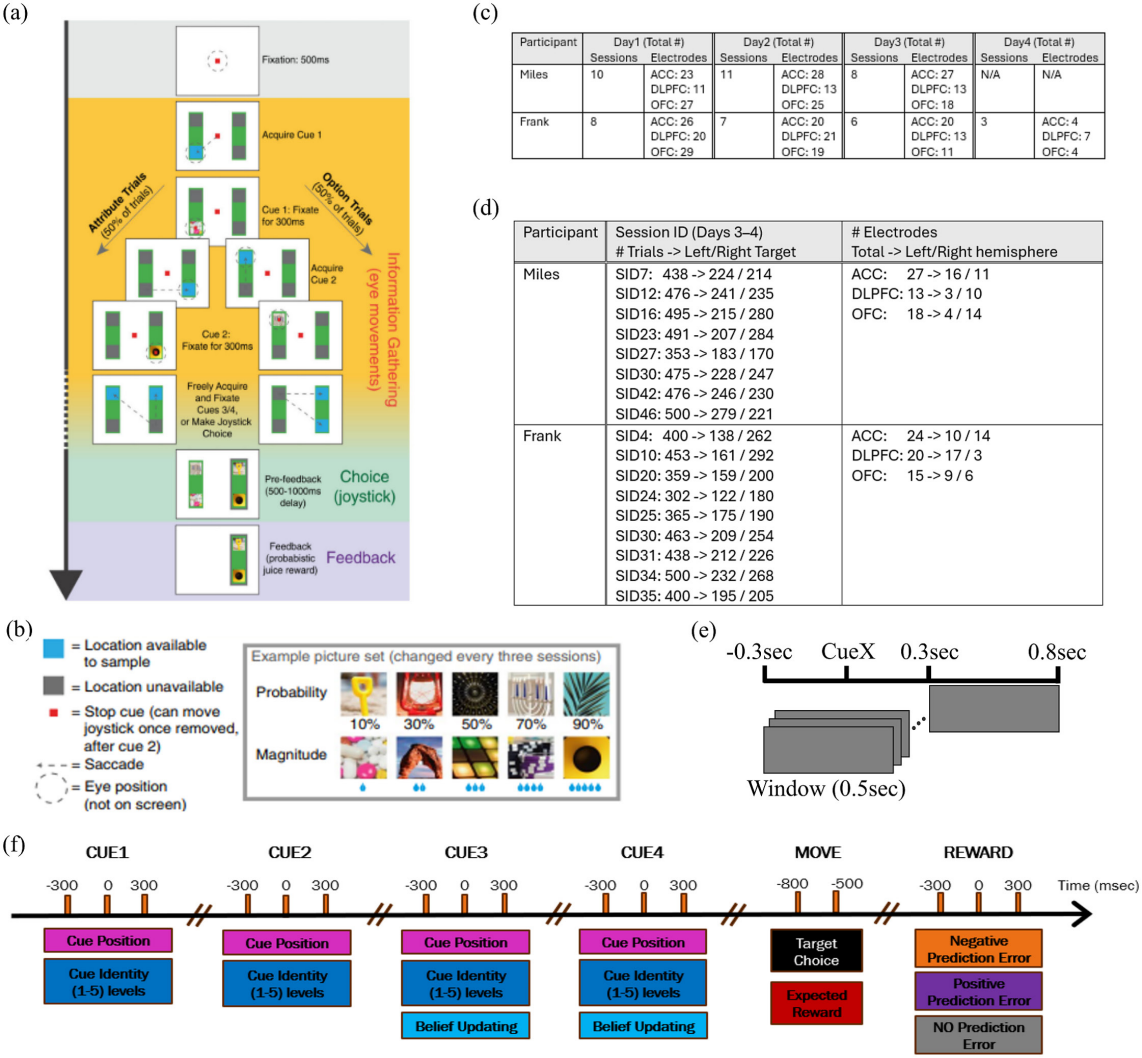
Task structure, cue sets, and recording summary. (a, b) Task schematic and cue set structure, adapted from Hunt, Malalasekera, de Berker, et al. (2018), showing sequential cue presentation and the associated reward probability and magnitude cues. (c) Summary of session and electrode counts for a given number of days using the same reward‐association picture set. (d) Summary of trial counts and electrode coverage for Day 3–4 sessions, by region and hemisphere for each participant. (e) Schematic example of the forward‐looking 500‐ms analysis window timing around a task event (cue shown as example). (f) Timeline of trial events and key cognitive variables analyzed at each stage, including cue‐driven preference revision, target choice, expected reward, and prediction error.

Global spectral analyses were restricted to Day 3–4 sessions to focus on periods of stable task performance. Population‐level decoding analyses were performed separately for each day (Days 1–3), with trials pooled across sessions within a given day to increase the number of available trials (Figure 1c,d). Sessions from the same day but with different cue‐reward associations were combined, and sessions with insufficient trial counts were excluded.

Because electrodes were inserted acutely each day, recording locations varied slightly across sessions. This approach allowed sampling across multiple sites within ACC, DLPFC, and OFC, providing broad coverage of the targeted prefrontal regions.

### 2.3 Behavioral Task

Animals performed a sequential reward‐guided decision‐making task in which up to four sequential visual cues conveyed probabilistic or value‐based information regarding two potential choice options (Figure 1a,b). Each cue revealed either the reward magnitude or the probability of the reward associated with a specific target (left or right), enabling the monkeys to accumulate and update their reward expectations over time. Animals integrated this information across cues to determine which target had the higher EV. Expected value was defined as follows: EV = probability × reward magnitude.

After cue exploration, the monkeys selected one of the two targets by moving a joystick in the corresponding direction. Reward delivery depended on the final cumulative EV of the chosen option.

The trials included two types: “Attribute” trials, in which monkeys fixated on Cue1 and Cue2 that presented the same attribute (either reward magnitude or probability) for both choice options; and “Options” trials, in which the first two cues provided different attributes (magnitude and probability) for the same option. After Cue2, the monkeys could freely sample additional cues (cues 3 and 4) before initiating a choice (Figure 1a; adapted from Hunt, Malalasekera, de Berker, et al. 2018). Reward feedback was delivered after movement completion with a fixed delay that differed between subjects (1000 ms in Miles and 500 ms in Frank).

Visual cues were linked to either the left or right choice target and signaled one of two attributes: reward magnitude or probability. Each cue set contained five discrete levels (10%–90% probability; 1–5 juice units) and was randomly assigned to represent one attribute per session (Figure 1b; adapted from Hunt, Malalasekera, de Berker, et al. 2018). Eye movements and joystick positions were monitored continuously throughout the trial.

For neural analyses, trials were segmented into task‐aligned epochs defined relative to key behavioral events, including cue onsets (cues 1–4), movement initiation, and reward delivery (Figure 1f).

Trials varied in cue value (magnitude vs. probability levels), spatial position (left vs. right), and whether new information supported or contradicted the currently preferred option (cue‐driven preference revision). Because trial type affects cue‐driven preference revision at Cue1 and Cue2 (attribute vs. option trials), this variable was analyzed only from Cue3 onward, where the regressor is comparable across trial types. By contrast, trial type is irrelevant for other variables such as cue level and cue position, which are intrinsic properties of each cue and can therefore be analyzed independently at each task event.

### 2.4 LFP Preprocessing and Feature Extraction

LFP signals were downsampled to 1 kHz and notch‐filtered at 50 Hz to remove line noise. Additional harmonic notch filters at 100 and 150 Hz were not applied because these frequencies fall within the Hγ band (50–150 Hz) used in the primary analyses; filtering them could remove neural signal components. A control analysis comparing preprocessing with a 50‐Hz notch filter alone versus additional harmonic notch filters at 100 and 150 Hz yielded highly similar results for both statistical modulation and decoding analyses (Figure S3), indicating that harmonic line noise did not influence the reported results.

A common average reference (CAR) was calculated by averaging the raw LFP signals across all channels in the session and then subtracting this mean from each channel's signal. This referencing was performed separately for each session, helping reduce spatially correlated noise and global artefacts.

To assess how neural activity evolved over time relative to key task events, we applied a moving window analysis to the preprocessed LFP signals (see Figure 1e for an example around a cue event). For each time point shown in the time‐resolved plots, we extracted features from a 0.5‐s forward‐looking window beginning at that time point and extending 500 ms into the future (i.e., [*t*, *t* + 0.5 s]). Consequently, the value assigned to time *t* reflects neural activity occurring throughout the subsequent 500‐ms interval rather than activity centered on *t*. For example, windows beginning before event onset may include neural activity occurring after the event. This approach was used consistently for both statistical analyses (e.g., assessing Hγ modulation relative to task variables) and decoding using support vector machines (SVMs). The sliding window moved in 50‐ms increments across task‐aligned epochs (cue onset, movement, and reward outcome), enabling fine‐grained temporal resolution of task‐related neural modulation while maintaining a consistent temporal alignment with ongoing task events.

Spectral power in the Hγ band (50–150 Hz), beta band (13–30 Hz), and theta band (4–8 Hz) was then extracted from each analysis window using the MATLAB multitaper method (*pmtm(), 3* sine tapers). We note that the 0.5‐s analysis window, while ideal for capturing the fast dynamics of the Hγ and beta bands, may be suboptimal for accurately capturing power in the lower frequency theta band. The limited window length may reduce the accuracy and stability of theta power, which should be considered when interpreting the results for this frequency band.

For statistical analyses, band power values were not log‐transformed or z‐scored, preserving absolute amplitude differences across conditions. For decoding analyses, the process did include normalization, as features were z‐scored within each training fold of the support vector machine (SVM) cross‐validation pipeline. Thus, decoding performance was not influenced by absolute amplitude differences across channels or sessions, and the use of absolute band power did not compromise classifier comparability.

### 2.5 Analysis of Task Variable Modulation

Our goal was to determine whether each task variable shows evidence of modulation in LFP activity, rather than to estimate partial contributions among variables within a unified model. Accordingly, we used single‐variable regressions to evaluate conceptually distinct hypotheses aligned to specific task epochs (e.g., cue level during cue periods, expected reward around movement and outcome contrasts at reward). Although some variables overlap temporally (e.g., around Cue3), the task design reduces correlations among regressors (Hunt, Malalasekera, de Berker, et al. 2018).

To assess robustness to potential residual correlations among task variables, we conducted control multivariate regression analyses in which cue‐driven preference revision, cue level, and cue position were entered simultaneously as predictors of Hγ power in the −300‐ to +300‐ms epoch around Cue3. For each channel, the minimum *p* value (pv) associated with the preference revision regressor within this interval was extracted, and false discovery rate correction (Benjamini–Hochberg, *α* = 0.05) was applied across channels within each subject and region.

Given the high‐dimensional mixed‐selectivity structure of prefrontal neural populations (Rigotti et al. 2013), the present univariate analyses should be interpreted as isolating task‐aligned components of neural activity rather than capturing the full representational dimensionality of the population code. Consistent with mixed selectivity, task‐related effects likely reflect overlapping representations of multiple task variables within a shared population.

### 2.6 Statistical Analysis

The statistical significance of the task‐variable modulation was assessed using linear regression. For each variable and time window, we fit a linear model relating Hγ power to the variable of interest across trials. Statistical significance was evaluated using the pv associated with the regressor of interest. For visualization, pvs were transformed to −log₁₀(pv) and used to summarize modulation strength across channels and regions.

### 2.7 Decoding Analysis

Linear SVM classifiers were trained on Hγ features to decode task variables across the task. Features were extracted from a 0.5‐s window aligned to a task event (e.g., cue onset, movement, or reward). Each classifier was evaluated using a fivefold cross‐validation scheme, and the decoding accuracy was computed as the mean classification accuracy across folds. Cross‐validation folds were defined at the trial level, and training and test sets were strictly separated within each fold. Feature normalization (z‐scoring) was performed using parameters computed exclusively from the training data and then applied to the held‐out test data, preventing information leakage across folds.

Class labels were balanced by subsampling equal trial counts per condition. For the multiclass decoding analyses (e.g., cue value with five levels), we implemented a one‐vs‐rest SVM strategy, in which each class was separately compared against all other classes combined. For each classification target, trial groupings were defined independently and trial order within each condition was randomized prior to decoding. Because decoding windows overlapped substantially (500‐ms window with 50‐ms step), adjacent time points should be interpreted as temporally smoothed trajectories rather than statistically independent estimates.

To estimate the decoding performance more robustly, the classification procedure was repeated 20 times using different random splits of training and testing data. We used 20 iterations because decoding accuracy and empirical chance estimates were stable across this number of repetitions. The average accuracy across the 20 iterations was used as the final decoding performance for each condition.

To generate a chance‐level baseline for comparison, the condition labels were randomly shuffled across trials, and the full decoding procedure was repeated 20 times on the shuffled data. The full decoding pipeline (including trial balancing, cross‐validation, and feature normalization) was repeated for each shuffled iteration. The resulting shuffled accuracy distribution was used to verify that observed decoding performance exceeded empirical chance levels; mean shuffled accuracy is shown in figures for reference.

Prior work from our group and others, using similar SVM‐based LFP decoding approaches, shows that decoding performance typically saturates with only a small subset of the most informative electrodes (Johnston et al. 2021, 2023). To assess statistical significance, observed accuracies were compared to empirical chance levels derived from label‐shuffled data. In addition, supplementary trial‐resampling analyses were conducted to generate region‐level statistics. For each cognitive variable, SVM decoding was repeated across 20 random trial subsets, and the resulting accuracy distributions were compared across electrode groupings (PFC subregions), with full statistics reported in Table S1.

### 2.8 Task Variables

The following classification targets were used in decoding analyses.

#### 2.8.1 Cue‐Driven Preference Revision

Cue‐driven preference revision was defined as trials in which newly revealed cue information reversed the currently preferred option based on cumulative EV. For cues 3–4 (evaluated only for left‐side cues; see Table S1 for right‐side and combined cues), we computed the cumulative EV for both options before and after the cue. If the side with higher EV reversed following the cue, the trial was labeled as a cue‐driven preference revision trial; otherwise, it was labeled as confirmation. A threshold of 0.1 EV units was applied to the preferred side to reduce label switching due to near ties. This operational definition captures cue‐driven changes in the currently preferred option and does not directly measure continuous latent belief states.

To test whether Hγ reflects graded evidence updating beyond categorical reversal, we examined trial‐by‐trial relationships between Hγ power and the magnitude of cue‐driven preference change at Cue3. Preference evidence was defined as EV_left − EV_right. Cue‐driven change (ΔPref) was computed as the Cue3–induced shift in this value, and |ΔPref| indexed revision magnitude. Hγ power was measured in the 50‐ to 550‐ms window following Cue3 onset. Linear regressions were performed separately for each channel.

Frank exhibited a consistent right‐choice bias across sessions (negative left–right choice difference; Figure S1), with left choices occurring less frequently. His error rates were also consistently higher when the left option was correct (Figure S2), suggesting a tendency to default to the right option under uncertainty. This behavioral bias could explain why decoding analyses, particularly for Frank, revealed stronger cue‐driven preference revision signals for left‐side cues, which required overriding his default choice tendency and may therefore have elicited stronger neural signatures of preference updating. Accordingly, we primarily focused our analyses on left‐cue trials, which more reliably differentiated this activity.

#### 2.8.2 Cue Position

The cue position (left vs. right) was determined based on the side of the screen to which the cue referred. The cues were labeled as left or right, depending on the spatial location of the corresponding target, regardless of their vertical screen position. This allowed us to isolate lateralized cue‐related modulation independent of layout.

#### 2.8.3 Cue Value Level

Each cue represented either reward probability or reward magnitude and was assigned one of five discrete levels defined by the task design (Hunt, Malalasekera, de Berker, et al. 2018).

For cue level decoding analyses, these levels were treated as ordinal labels (1–5), where 1 corresponded to the lowest value and 5 to the highest value.

For analyses involving EV, cue levels were mapped to normalized numerical values. Probability levels were represented as [0.1, 0.4, 0.5, 0.7, 0.9], and magnitude levels were represented using normalized values [0.15, 0.35, 0.55, 0.75, 0.95]. These values were used to compute EV for the preference revision and expected reward analyses.

#### 2.8.4 Expected Reward

To assess reward expectation at the time of movement, we computed the final EV of the chosen target by multiplying its cumulative probability and magnitude. These values were discretized into three bins (low, medium, and high). For binary decoding, only the trials in the lowest and highest bins were retained to maximize class separability.

#### 2.8.5 Target Choice

The target choice was determined based on the direction of joystick movement. The left and right choices were labeled 1 and 2, respectively, and were used for decoding around the movement epoch.

#### 2.8.6 Reward Outcome and Prediction Error

In this task, EV is defined as magnitude × probability. Although reward magnitude varies from one to five drops and EV is well defined, outcome events in this task are categorical (reward vs. omission), and surprise arises from combinations of outcome and probability. Consequently, prediction‐error–related responses arise from combinations of outcome and probability, and probability and surprise remain partially confounded.

Empirically, prediction‐error–related responses were best reflected by three categorical contrasts:

1. Negative Prediction Error (Negative PE): High‐probability trials (≥ 0.7) with omitted rewards were contrasted with low‐probability trials (≤ 0.5) in which rewards were also omitted. This contrast captures outcome‐related responses associated with unexpected versus expected reward omission and may reflect a combination of surprise and reward probability.
2. Positive Prediction Error (Positive PE): Low‐probability trials (≤ 0.5) with delivered rewards were contrasted with high‐probability trials (≥ 0.9) in which rewards were also delivered. This contrast captures outcome‐related responses associated with unexpected versus expected reward delivery and may reflect a combination of surprise and reward probability.
3. No Prediction Error (No PE): Trials in which reward outcomes matched expectations (high‐probability rewarded versus low‐probability unrewarded) were used as controls to characterize the dominant reward‐evoked response. This contrast primarily reflects reward outcome valence together with differences in reward probability.

These contrasts reflect outcome–probability interactions defined by the task structure rather than formal model‐derived signed prediction errors. Only trials with complete cue and outcome information were included in the analysis.

### 2.9 Multisession Trial Aggregation for Decoding

To construct population‐level decoding datasets spanning a broader range of recording sites, we aggregated trials from multiple sessions. For each classification condition (e.g., left vs. right target choice), trials were selected independently from each session and stacked to form a unified decoding dataset. The trials were matched across sessions based on the condition labels, timing constraints, and minimum premovement intervals. To ensure balanced class sizes and avoid classifier bias, an equal number of trials was included per condition (e.g., equal numbers of left and right choices), with a fixed maximum number drawn per group. Trials were only included if sufficient time was available between the aligned event (e.g., Cue4) and the next event (movement) to avoid contamination from movement onset. The LFP data for each selected trial were extracted and aligned to the relevant event, and each trial's LFP signal was the common average referenced within the session before being concatenated across channels and sessions. This procedure allowed us to create pooled decoding matrices spanning multiple sessions, preserving label integrity while reducing contamination from adjacent task events. Because cue‐reward mappings differed across sessions, decoding targets were defined using task‐level variables (e.g., relative EV, preference revision) rather than specific stimulus identities, ensuring that decoding reflected abstract task variables rather than fixed cue‐image associations.

To assess the robustness of pooled decoding results to session composition, we performed a leave‐one‐session‐out analysis (Table S2). Because electrodes were sampled acutely and varied in number and anatomical location across sessions, some variability in decoding performance was expected across session exclusions. The leave‐one‐session‐out analysis was therefore used to assess whether pooled decoding results were robust to changes in session composition and whether decoding performance remained above empirical chance following exclusion of individual sessions. The resulting decoding accuracies are summarized in Table S2 and indicate that the principal decoding effects were not dependent on any single recording session.

## 3 Results

### 3.1 High‐Gamma Is the Most Informative Frequency Band Across Cognitive Variables

**FIGURE 2:**
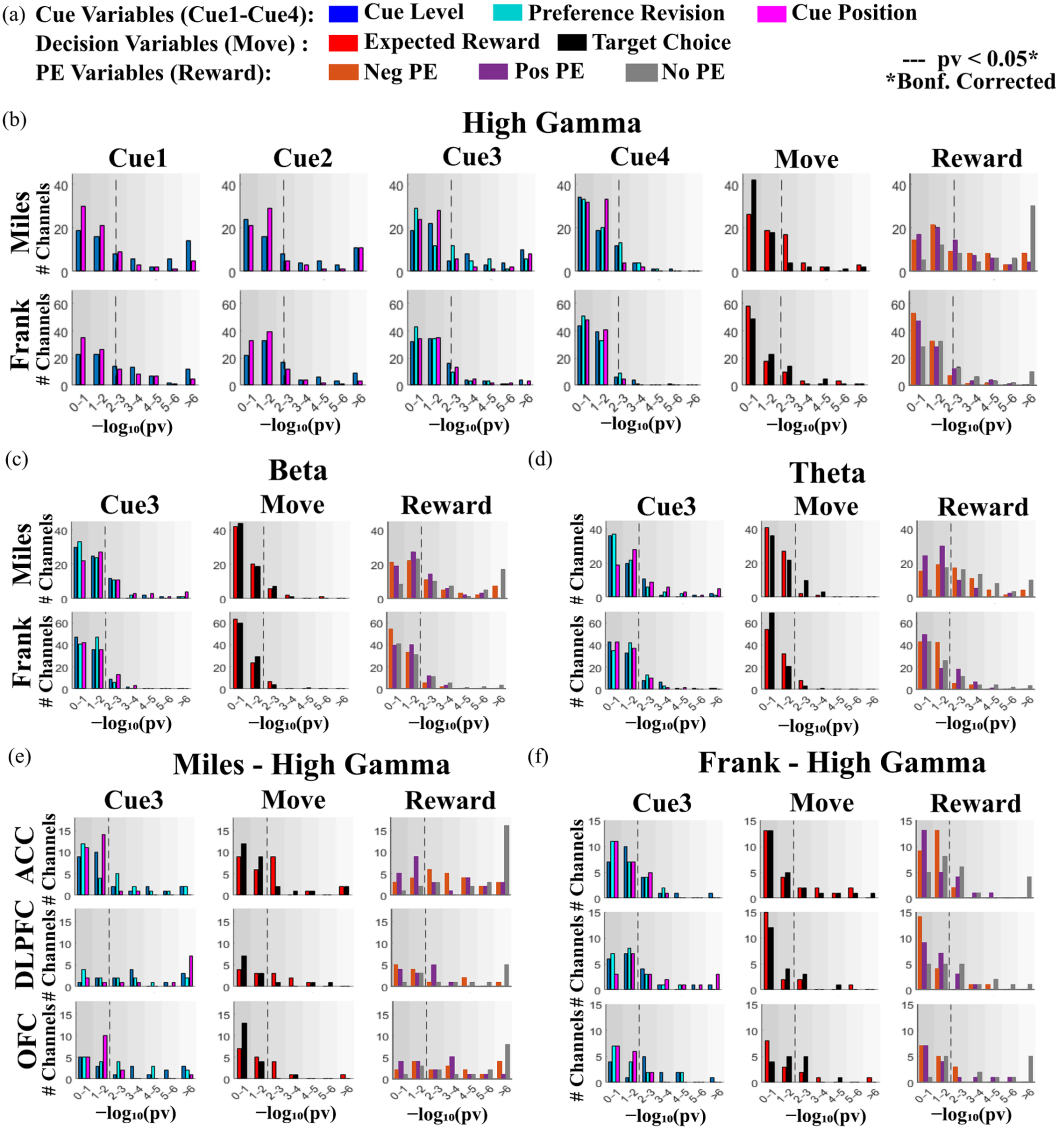
Task‐variable LFP modulation across time points, frequency band, and subjects. (a) Legend for task variables grouped by relevance to task phase: cue‐related variables (blue: cue level; magenta: cue position; cyan: cue‐driven preference revision) are evaluated during Cue1–Cue4, decision‐related variables (red: expected reward; black: target choice) are evaluated prior to movement, and prediction error variables (orange: negative prediction error; purple: positive prediction error; gray: no prediction error) are evaluated during the reward period. (b) Histograms of channel‐level Hγ modulation strength by each task variable in subject Miles (top row) and Frank (bottom row) across time points. (c–d) Same format as (b) but for beta (c) and theta (d) power. (e, f) Distribution of channel‐level Hγ modulation by task variable for Cue3, move, and reward in subject Miles (e) and Frank (f), separated by prefrontal region: ACC, DLPFC, and OFC. Bars indicate the number of channels with a given −log₁₀(pv), grouped in bins of width 1; the final bin (> 6) includes all more strongly modulated channels. The dashed line indicates the Bonferroni‐corrected significance threshold: pv = 0.05/13 for Cue1–Cue4 and reward, pv = 0.05/7 for move. Table S3 provides the corresponding normalized proportions (%) and exact counts of Bonferroni‐significant channels relative to the total number of recorded channels within each region for all task variables.

To assess population‐level modulation associated with task variables, we tested whether LFP power in the Hγ, beta, and theta bands (see methods) was significantly modulated by cue‐related variables (cue level, cue position, and cue‐driven preference revision) during Cue1–Cue4; decision variables (target choice and expected reward) prior to movement; and outcome‐related contrasts (negative PE, positive PE, no PE) around the reward event (Figure 2a–d). Global spectral analyses used Days 3 and 4 sessions to focus on periods expected to have the most stable behavior. The modulation strength for each channel was determined by the maximum −log₁₀(pv) across the task event windows. Figure 2b aggregates these channel‐level results to summarize spectral modulation patterns across PFC.

Note that, in our dataset, the number of electrodes varies substantially across regions. Because differences in electrode sampling may influence visual interpretation of absolute channel counts, we additionally report both the exact number and proportion (%) of Bonferroni‐significant channels relative to the total recorded channels within each region in Table S3. Presenting both the full modulation distributions and the normalized proportions provides a more balanced and transparent assessment of regional modulation patterns across prefrontal areas. Formal inferential comparisons were based on the decoding analyses reported in Table S1.

We observed widespread task‐related modulation of Hγ power, particularly during Cue1–Cue3, move, and reward, the task phases associated with cue evaluation, action execution, and reward processing (Figure 2b). Cue level and cue position produced widespread Hγ modulation across Cue1–Cue3, indicating early differentiation between cue features during sequential evaluation. In the premovement epoch, Hγ was strongly modulated by target choice and expected reward, reflecting decision formation and reward anticipation. Theta showed weaker and more variable modulation in this epoch (Figure 2d), whereas beta effects were sparse across task variables (Figure 2c).

Regional grouping of electrodes (ACC, DLPFC, and OFC) revealed distinct but overlapping spatial patterns (Figure 2e–f). Cue‐driven preference revision engaged channels across all three regions. Although ACC contained a relatively large number of significantly modulated channels, normalization by regional electrode count revealed substantial proportional involvement of DLPFC and OFC. DLPFC showed prominent modulation for cue position, while cue level differentiation was distributed across ACC, DLPFC, and OFC, with the strongest proportional modulation varying across cue epochs and subjects.

Given the stronger and more consistent modulation observed in the Hγ band, all subsequent analyses focused on Hγ activity.

### 3.2 Temporal Dynamics of Task‐Variable High‐Gamma Modulation Across Brain Regions

**FIGURE 3:**
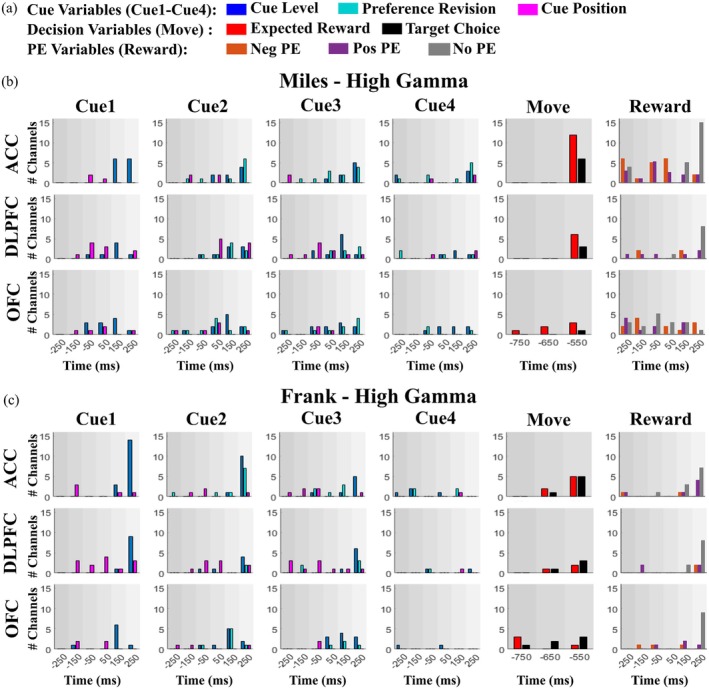
Temporal dynamics of task‐variable modulation in Hγ activity across frontal regions. (a) Legend for task variables (same as Figure 2). (b, c) Temporal profiles of significantly modulated channel counts for subject Miles (b) and Frank (c), grouped by region (ACC, DLPFC, and OFC) and plotted in 200‐ms time bins (x‐axis: time relative to cue, movement, or reward onset; y‐axis: channel count). Table S3 provides the corresponding normalized proportions (%) and exact counts of Bonferroni‐significant channels relative to the total number of recorded channels within each region for all task variables. *Note:* Time points represent the start of a forward‐looking 500‐ms window [*t*, *t* + 500 ms] and therefore include neural activity occurring throughout the subsequent 500‐ms interval rather than activity centered on the labeled time.

To examine the temporal dynamics of task‐related modulation of Hγ activity, we analyzed the number of significantly modulated channels across time, grouped by brain region (ACC, DLPFC, and OFC) and task epoch (Cue1–Cue4, move, and reward; Figure 3a).

Cue‐related variables produced robust Hγ modulation across all three regions (Figure 3b,c for Miles and Frank). Hγ modulation was observed throughout the cue‐aligned epochs and persisted for several hundred milliseconds across Cue1–Cue3. Cue level and cue position produced the most prominent modulation across cue epochs, while Cue‐driven preference revision also engaged channels across ACC, DLPFC, and OFC, with variable temporal profiles across subjects and regions.

Prior to movement onset (−800 to −500 ms), we observed significant Hγ modulation by expected reward and target choice across ACC, DLPFC, and OFC for both subjects. This temporal window corresponds to the decision phase preceding action initiation. Although modulation was distributed across regions, ACC and DLPFC activity tended to peak in windows immediately preceding movement onset, whereas OFC modulation often emerged earlier and was more temporally distributed across the premovement interval.

Following movement, Hγ modulation was predominantly associated with reward feedback processing (see Methods). This activity was strongly modulated in the no PE contrast, reflecting sensitivity to reward outcome. Positive and negative PE contrasts, corresponding to unexpected versus expected reward delivery and omission, respectively, also elicited Hγ modulation in ACC, DLPFC, and OFC (Figure 3b,c; reward epoch). This distributed pattern suggests that outcome‐related and outcome–probability interaction contrasts engage multiple prefrontal regions during feedback processing.

Overall, these results revealed distinct temporal profiles of cue, decision, and feedback‐related modulation in Hγ activity across the prefrontal regions. Although certain task variables showed stronger modulation in particular regions or epochs, substantial overlap across ACC, DLPFC, and OFC was observed, consistent with distributed and mixed‐selective representations of task‐relevant information across PFC.

### 3.3 Cue Feature‐Related Modulation in High‐Gamma Across PFC Regions

**FIGURE 4:**
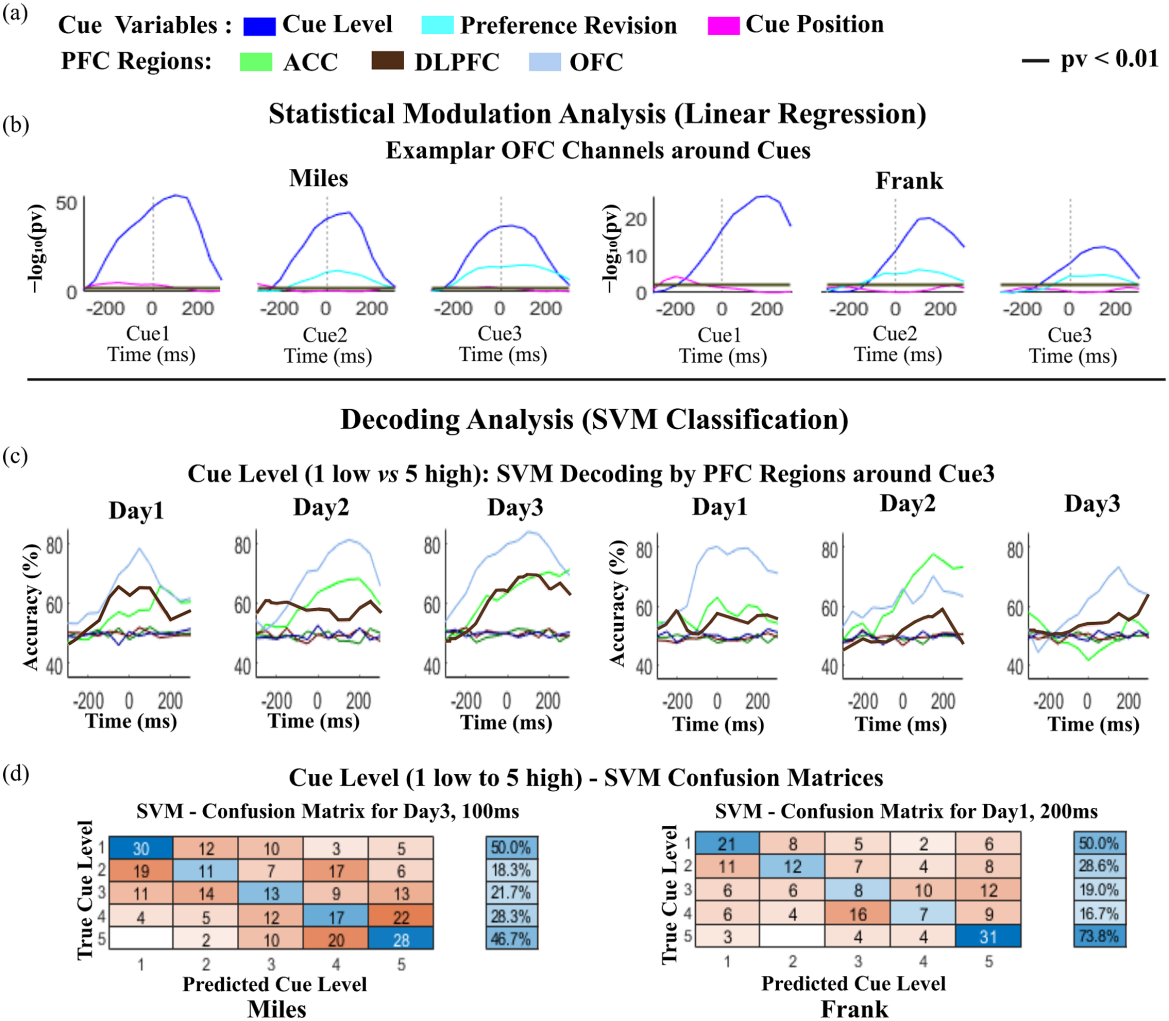
Region‐specific modulation and decoding of cue level across PFC subregions. (a) Legend for task variables: cue‐related variables (blue: cue level; cyan: cue‐driven preference revision; magenta: cue position) and PFC subregions. (b) Exemplar channels from Miles (left) and Frank (right) showing strong modulation for cue level around Cues 1–3 trial epochs. (c) SVM linear decoding performance (fivefold cross‐validation) for Hγ activity using pooled pseudo‐population datasets constructed from sessions on Days 1–3 and channels from ACC (green), DLPFC (brown), and OFC (teal). Dark lines indicate chance‐level performance (shuffled labels). (d) Confusion matrices of SVM decoding for cue levels (five‐level decoding: 1 = lowest value/probability; 5 = highest value/probability), based on pooled channels from all PFC regions. Left: Day 3 decoding for Miles; right: Day 1 decoding for Frank. Blue columns indicate correct classification rates per level. *Note:* Time points represent the start of a forward‐looking 500‐ms window [*t*, *t* + 500 ms] and therefore include neural activity occurring throughout the subsequent 500‐ms interval rather than activity centered on the labeled time.

**FIGURE 5:**
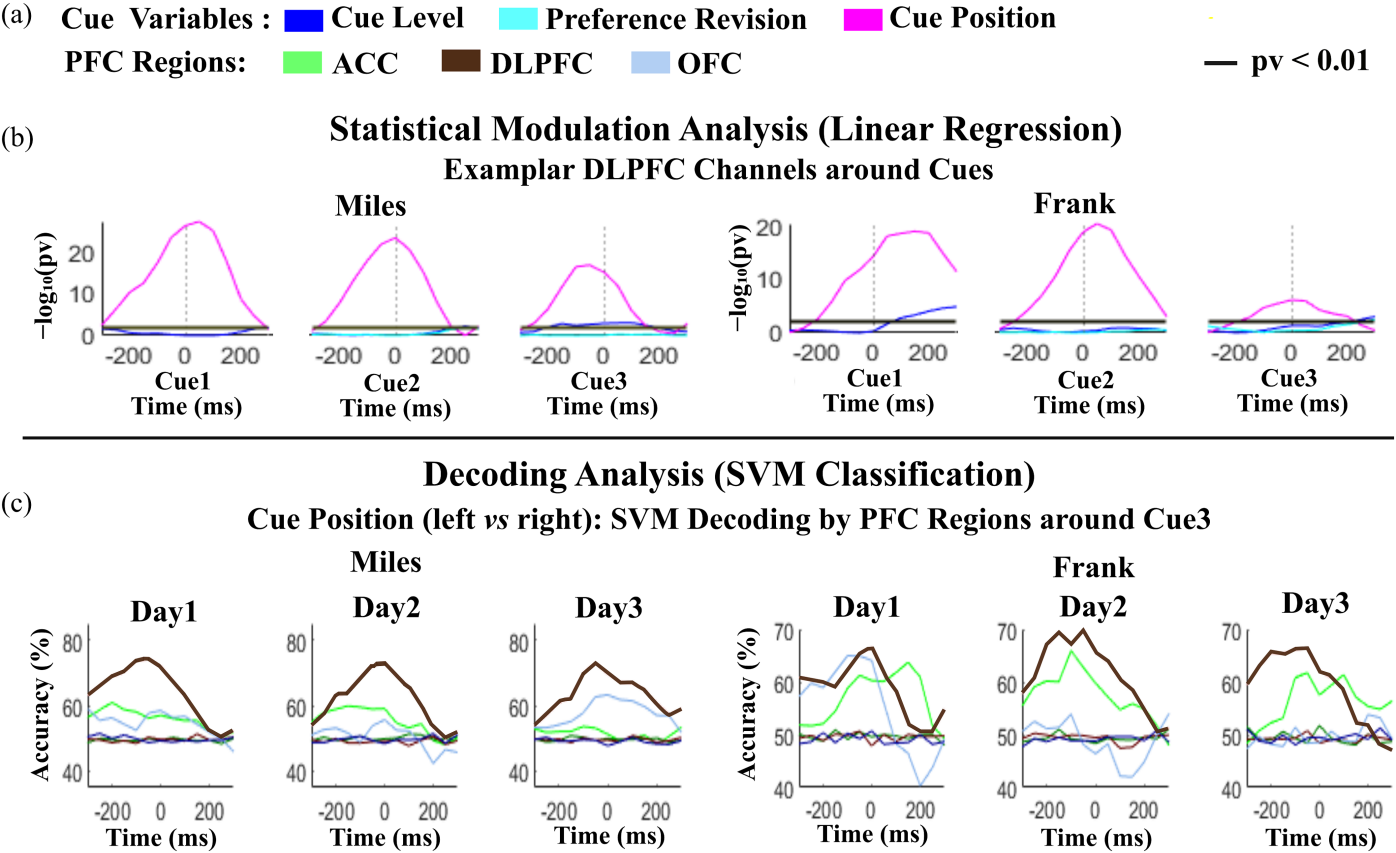
Region‐specific modulation and decoding of cue position across PFC subregions. (a) Legend for task variables: cue‐related variables (blue: cue level; cyan: cue‐driven preference revision; magenta: cue position) and PFC subregions. (b) Exemplar channels from Miles (left) and Frank (right) showing strong Hγ modulation for cue position (left vs. right cues) around cues 1–3 trial epochs. (c) SVM linear decoding performance (fivefold cross‐validation) for Hγ activity using pooled pseudo‐population datasets constructed from sessions on Days 1–3 and channels from ACC, DLPFC, and OFC. Dark lines indicate chance‐level performance (shuffled labels). *Note:* Time points represent the start of a forward‐looking 500‐ms window [*t*, *t* + 500 ms] and therefore include neural activity occurring throughout the subsequent 500‐ms interval rather than activity centered on the labeled time.

To examine region‐specific differentiation of cue‐related variables, we analyzed Hγ activity in ACC, DLPFC, and OFC. Single‐channel analyses revealed robust modulation by cue level and cue position, with distinct temporal profiles (Figures 4a and 5a).

Exemplar OFC channels (Figure 4b) showed strong cue level modulation during cue‐aligned epochs, whereas some DLPFC channels (Figure 5b) responded strongly to cue position. While each example emphasized one dominant variable, both exhibited weaker modulation of other features, consistent with mixed selectivity.

Population‐level SVM decoding (Figures 4c and 5c) using pooled trials from Days 1–3 showed that cue level (high vs. low level) was robustly decodable from OFC in both animals, even during early learning. In contrast, cue position decoding was strongest in DLPFC, particularly in Miles. ACC contributed to spatial decoding in Frank but less so in Miles, indicating subject‐specific variability. Dark traces denote shuffled‐label chance performance. Results were consistent with the region‐level statistical comparisons reported in Table S1.

Confusion matrices for five‐level cue level decoding at Cue3 (Figure 4d) revealed stronger discrimination of extreme values (level 1 = low to level 5 = high) compared to intermediate levels, which were more frequently confused. This pattern indicates stronger differentiation for extreme cue values.

Together, these results reveal both variable‐ and region‐specific Hγ differentiation between cue features across PFC, with OFC showing the strongest cue level differentiation, DLPFC exhibiting the strongest cue position differentiation, and ACC contributing in a subject‐dependent manner within a distributed coding architecture.

### 3.4 Cue‐Driven Preference Revision in High‐Gamma Activity Across PFC Regions

To examine how Hγ activity differentiates trials requiring evidence‐driven preference revision during cue processing, we analyzed individual channels across PFC subregions (ACC, DLPFC, and OFC) in both subjects. Cue‐driven preference revision was defined as trials in which the current cue reversed the previously preferred option based on cumulative EV (i.e., when the relative EV difference changed sign). This operational definition captures decision‐level policy revision rather than continuous latent belief dynamics.

Because Frank exhibited a consistent rightward choice bias (Figures S1 and S2), revision signals were strongest and most reliable for left‐cue trials, which required overriding this default tendency. Accordingly, analyses focused on left‐side cues, where decoding was most consistent across animals; the results for right‐side and combined cues are provided in Table S1.

**FIGURE 6:**
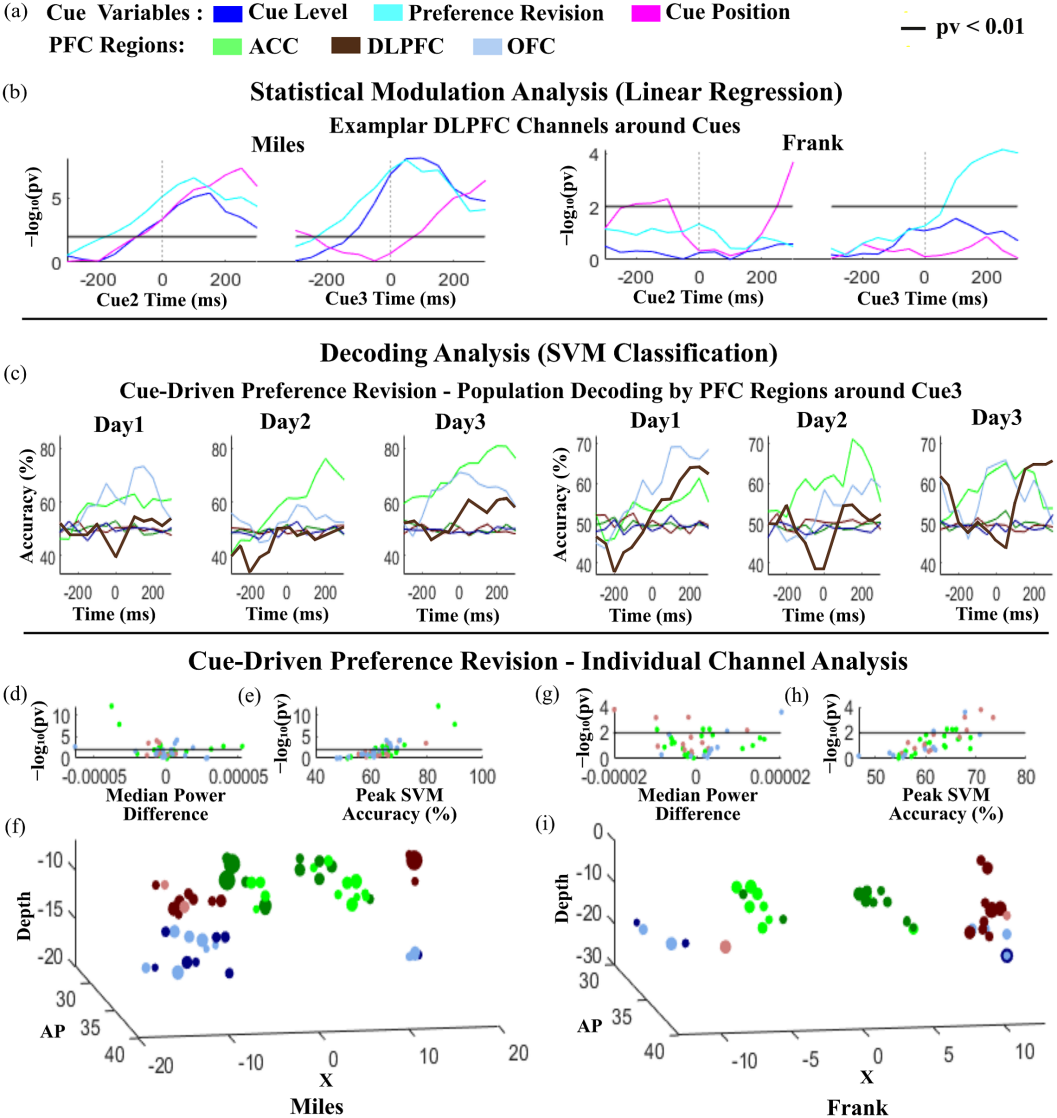
High‐gamma modulation and decoding of cue‐driven preference revision in left cue trials in PFC subregions. (a) Legend for cue variables and PFC subregions. (b) Exemplar channels from Miles (left) and Frank (right) showing task‐related modulation of Hγ power around Cue2 and Cue3, with statistical strength shown as −log₁₀(pv). (c) SVM decoding accuracy for cue‐driven preference revision around Cue3 across sessions from Days 1–3, plotted separately by subject and PFC subregion. Darker traces represent chance‐level from shuffled labels. (d, g) Scatterplots of statistical significance (−log₁₀(pv)) versus median Hγ power difference between cue‐driven preference revision and Confirmation trials. (e, h) Scatterplots of channel‐level peak decoding accuracy and statistical significance (−log₁₀(pv)) for the same contrasts. (f, i) Anatomical distribution of recorded channels in PFC, showing electrode depth (Y‐axis), anterior–posterior position (AP), and lateral (X) positions. Marker size reflects peak decoding accuracy; color corresponds to PFC subregion (ACC: green, DLPFC: brown, OFC: teal), with darker shades indicating higher median Hγ power during cue‐driven preference revision than confirmation trials. *Note:* Time points represent the start of a forward‐looking 500‐ms window [*t*, *t* + 500 ms] and therefore include neural activity occurring throughout the subsequent 500‐ms interval rather than activity centered on the labeled time.

Figure 6a illustrates the color scheme used for cue‐related variables and PFC subregions. Exemplar channels from both animals (Figure 6b) demonstrated strong modulation for cue‐driven preference revision (cyan traces) aligned to Cue2 (where trial type may influence the observed modulation) and Cue3, with statistical strength visualized as −log₁₀(pv), demonstrating temporally specific Hγ sensitivity to cue‐driven value changes.

Linear SVM decoding of preference‐revision versus confirmation trials revealed above‐chance differentiation across regions (Figure 6c), with ACC often exhibiting the strongest decoding performance across days, particularly in Miles. The maximum accuracy reached 80.9% in Miles (ACC) and 71.1% in Frank (ACC). As detailed in Table S1, decoding was weaker for right‐cue trials and when pooling both cues, consistent with behavioral side bias.

We then analyzed preference‐reversal sensitivity at the single‐channel level using data from Day 3 sessions. Scatterplots of statistical significance (−log₁₀(pv)) versus the median Hγ power difference (Figure 6d for Miles; Figure 6g for Frank) revealed diverse patterns across subregions. Negative median differences corresponded to stronger Hγ activity during update preference relative to no update trials, whereas positive values reflected the opposite pattern. A bias toward negative values was most evident in the DLPFC of both animals. In contrast, ACC and OFC showed more balanced distributions. Thus, DLPFC showed a stronger bias toward update‐related modulation, whereas ACC and OFC displayed greater heterogeneity in response direction.

Channel‐level decoding performance varied substantially across recording sites (Figure 6e for Miles; Figure 6h for Frank). While many channels exhibited near‐chance decoding, a subset achieved markedly higher accuracies and also tended to show stronger task‐related modulation. These distributions provide a compact visualization of channel‐level decoding performance and statistical modulation strength, and complement the anatomical maps shown in Figure 6f,i.

Anatomical mapping of recorded channels (Figure 6f for Miles; Figure 6i for Frank) revealed localized distributions of revision‐modulated sites. Marker size reflects peak decoding accuracy, whereas color corresponds to PFC subregion (ACC: green, DLPFC: brown, OFC: teal). Within each subregion color, darker shades indicate negative median Hγ differences (greater Hγ activity during update preference relative to no update trials), whereas paler shades indicate positive median differences (greater Hγ activity during no update relative to update preference trials), as shown in Figure 6d,g.

In both Miles and Frank, visual inspection of the anatomical distributions suggested localized concentrations of high‐decoding channels within ACC and DLPFC. These maps suggest that revision‐related Hγ effects are spatially structured within PFC, although regional differences in electrode coverage may influence the apparent density of mapped channels. Overall, the observed distributions indicate subject‐ and region‐specific variability in revision‐related modulation patterns.

Across regions, a subset of channels exhibited significant relationships between Hγ power and cue‐driven preference change (ΔPref; see Methods). After false discovery rate (FDR) correction (*α* = 0.05), graded effects were observed in a minority of channels and varied by subject and region (Tables S4 and S5). Scaling with |ΔPref| was more prevalent than signed ΔPref effects, which were sparse and did not consistently survive correction. Effect sizes were modest (median partial *R* ^2^ < 0.01), indicating small but reliable graded modulation in select recording sites.

To assess whether these effects were independent of other cue variables, we performed a multivariate regression including preference revision, cue level, and cue position (see Methods). A subset of channels remained significantly modulated by preference revision after FDR correction (Tables S6 and S7), although regional prominence varied by subject.

Together with the univariate findings, these results indicate that cue‐driven preference revision effects are detectable across prefrontal regions and are not fully explained by cue level or cue position, although their regional prominence varies depending on model specification.

### 3.5 Decision‐Related Modulation of Choice and Value in High‐Gamma

**FIGURE 7:**
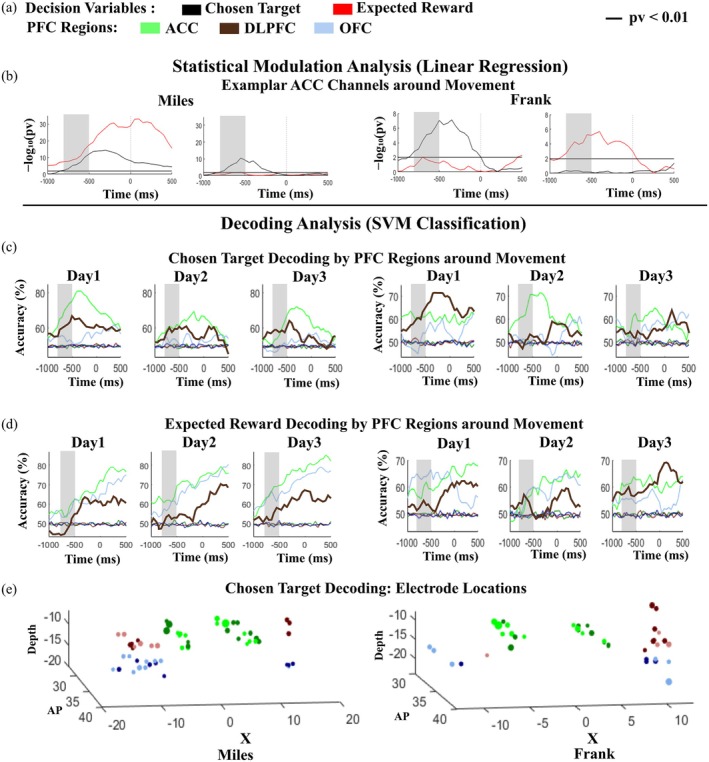
Decoding of target choice and expected reward from PFC Hγ activity across days. (a) Legend showing task variables and color codes. (b) Two exemplar channels per animal (Miles left, Frank right) showing trial‐aligned statistical modulation (−log₁₀(pv)) of Hγ activity by task variables during the movement epoch. Decoding time points represent the start of a 0.5‐s window. (c) SVM decoding accuracy (fivefold cross‐validation) for target choice (left vs. right) using pooled pseudo‐population datasets constructed from sessions on Days 1–3 and channels from PFC subregions. (d) SVM decoding accuracy (fivefold cross‐validation) for expected reward (high vs. low; medium trials excluded) using pooled pseudo‐population datasets constructed from sessions on Days 1–3 and channels from PFC subregions. (e) Anatomical distribution of chosen target decoding performance across all channels for Miles (left) and Frank (right). Marker size reflects decoding accuracy; color denotes region (ACC: green, DLPFC: brown, OFC: teal), with darker shades indicating higher Hγ power for the chosen target. *Note:* Time points represent the start of a forward‐looking 500‐ms window [*t*, *t* + 500 ms] and therefore include neural activity occurring throughout the subsequent 500‐ms interval rather than activity centered on the labeled time.

To examine decision‐related signals in Hγ activity, we analyzed target choice and expected reward around movement initiation (Figure 7). Animals integrated probabilistic cue information to select a target. We examined both single‐channel modulation and population‐level SVM decoding of target choice and expected reward across three learning days.

Exemplar ACC channels in both animals showed clear task‐related Hγ modulation in movement‐aligned windows (Figure 7b). Expected reward signals displayed diverse temporal dynamics: some channels discriminated high versus low reward in early premovement windows (starting 800–500 ms before movement), whereas others ramped gradually and peaked in windows spanning movement onset. Target choice modulation similarly increased across premovement windows and peaked in windows spanning movement onset, indicating that a subset of ACC channels progressively differentiated upcoming choices before action selection.

To quantify predictive strength, we trained linear SVM classifiers to decode left versus right target choices using Hγ power extracted from channels grouped by PFC subregion. In both Miles and Frank (Figure 7c, left and right), the decoding accuracy was above chance on Day 1 and remained robust through Days 2 and 3. During the decision window, ACC generally exhibited the strongest decoding, with additional contributions from DLPFC near movement onset.

Expected reward decoding (high vs. low) was also reliable across days (Figure 7d), with strongest performance in ACC and OFC ramping through the movement‐aligned epoch. DLPFC decoding showed modest improvement across days during the decision‐making phase, particularly in Frank.

Spatial mapping of peak channel‐level decoding during the decision window revealed localized concentrations of high‐decoding channels within ACC in both animals, consistent with region‐level decoding results (Figure 7e, left and right). Overall, decision‐related Hγ modulation was distributed across PFC, with target choice decoding strongest in ACC and expected reward decoding most prominent in ACC and OFC.

### 3.6 Frontal High‐Gamma Activity Reflects Reward Outcome and Prediction Errors

**FIGURE 8:**
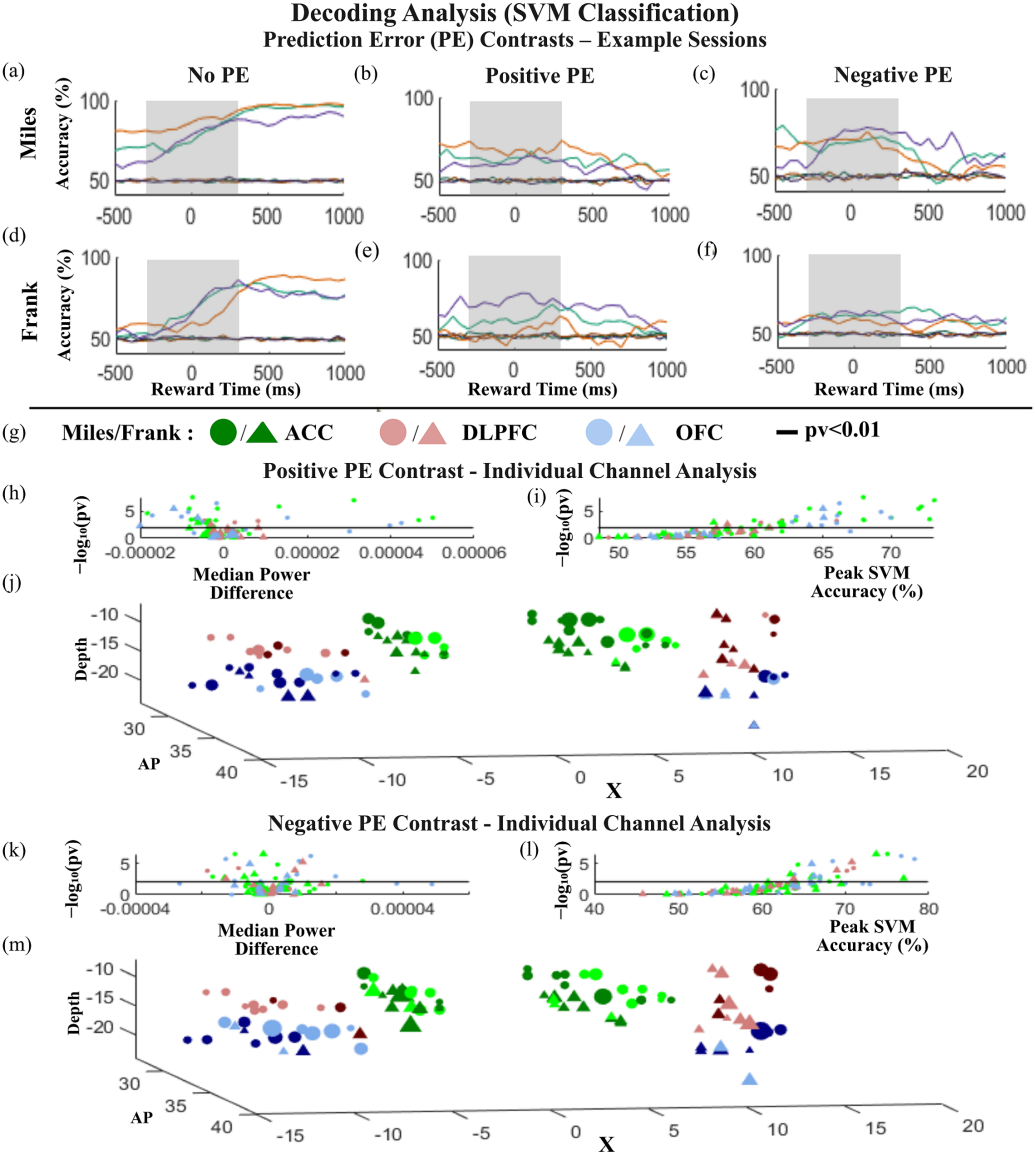
Frontal Hγ activity differentiates reward outcome and PE contrasts. (a–f) SVM decoding accuracy (fivefold cross‐validation) over time, showing results from all PFC channels in three exemplar sessions per animal. Panels show decoding for: (a, d) no PE: rewarded high‐probability versus nonrewarded low‐probability trials (Miles and Frank, respectively). (b, e) positive PE: low‐probability rewarded versus high‐probability rewarded trials (Miles and Frank, respectively). (c, f) negative PE: high‐probability unrewarded versus low‐probability unrewarded trials (Miles and Frank, respectively). (g) Legend showing PFC subregion colors (ACC: green, DLPFC: brown, OFC: teal) and subject markers (circles: Miles, triangles: Frank). (h, k) Relationship between median Hγ power difference and statistical significance (−log₁₀(pv)) across channels for positive and negative PE contrasts. (i, l) Scatterplots of channel‐level peak decoding accuracy and statistical significance (−log₁₀(pv)) for the same contrasts. (j, m) Anatomical distribution of decoding performance across channels using Day 3 sessions. Marker size reflects decoding accuracy; marker shape and color follow the legend in (g), with darker shades indicating channels where median Hγ power was higher for unexpected versus expected outcomes. *Note:* Time points represent the start of a forward‐looking 500‐ms window [*t*, *t* + 500 ms] and therefore include neural activity occurring throughout the subsequent 500‐ms interval rather than activity centered on the labeled time.

Prediction error (PE) contrasts (negative PE, positive PE, no PE) were defined as described in Methods (Section 2.8.6), and analyses focused on Day 3 sessions when behavior was stable. The time‐resolved decoding analyses shown in Figure 8a–f were performed using pooled PFC channels, with each line representing one session from each subject. Region‐specific time‐resolved decoding analyses are provided in Figure S4, whereas pooled regional decoding statistics at selected analysis windows are summarized in Table S1.

No PE decoding was consistently high across sessions in both animals (Figure 8a,d for Miles and Frank, respectively). As both trial types were expected based on cue probabilities, these results suggest that frontal Hγ activity robustly differentiates rewarded from non‐rewarded outcomes, even in the absence of surprise.

In the positive PE condition (Figure 8b,e), decoding remained above chance but was more variable across sessions, with higher performance in Miles than Frank. Channel‐level analyses (Figure 8h–i) revealed substantial variability in decoding performance across recording sites, with a relatively small subset of channels achieving notably higher accuracies than most recorded channels. These panels facilitate identification of highly informative channels contributing to the anatomical distribution shown in Figure 8j. Highly informative channels were observed across all three regions, although many of the highest‐performing sites were located in ACC and OFC. Region‐specific decoding traces (Figure S4) and pooled regional decoding analyses (Table S1) indicated relatively stronger positive PE decoding in OFC across subjects, whereas ACC and DLPFC contributions varied between animals.

In the negative PE condition (Figure 8c,f), decoding exceeded chance but was generally weaker and more variable than in the other outcome contrasts. Channel‐level analyses (Figure 8k,l) similarly showed substantial variability in decoding performance across recording sites, with a subset of channels achieving high decoding accuracies across ACC, DLPFC, and OFC. These panels facilitate identification of highly informative channels contributing to the anatomical distribution shown in Figure 8m. Anatomical mapping revealed informative individual channels in all three regions, although their density and spatial distribution varied across subjects. Consistent with the region‐specific decoding traces shown in Figure S4, pooled regional decoding analyses at the selected 300‐ms postreward window (Table S1) revealed subject‐dependent regional differences, with the highest decoding accuracy observed in DLPFC for Frank and OFC for Miles during the negative PE condition.

Although the strongest decoding emerged for reward versus no reward (no PE), unexpected reward delivery and omission were also detectable across ACC, DLPFC, and OFC. Individual‐channel analyses identified highly informative channels across ACC, DLPFC, and OFC, whereas pooled regional decoding analyses revealed subject‐dependent regional differences across prediction error conditions.

## 4 Discussion

This study demonstrated that Hγ activity in the PFC shows reliable modulation related to multiple task‐defined variables during reward‐guided decision‐making, including cue level and location, cue‐driven preference revision, target choice, expected reward, and outcome‐related contrasts. Decoding performance reflects the presence of discriminative information in the population rather than direct evidence of explicit encoding of task variables. Hγ power exhibited stronger and more consistent modulation than lower‐frequency bands, particularly during cue evaluation and movement planning, making it well‐suited for tracking task‐defined decision variables with high temporal precision. This aligns with prior research linking Hγ and broadband high‐frequency activity to local cortical computation and ensemble spiking (Buzsáki et al. 2012; Manning et al. 2009) and extends its functional relevance to decision‐related variables such as preference revision and outcome evaluation (Hunt et al. 2015).

During cue viewing, Hγ signals differentiated both cue level and cue position. Cue level decoding was strongest in the OFC, although significant modulation was also observed in ACC and DLPFC. This pattern is broadly consistent with prior studies implicating OFC in representing stimulus‐value associations and value‐related cue information (Padoa‐Schioppa and Assad 2006). A different pattern emerged for cue position, which showed the strongest Hγ differentiation in DLPFC, although significant effects were observed across ACC, DLPFC, and OFC. This finding is consistent with the established role of DLPFC in spatial working memory and spatial attention (Miller et al. 1996; Buschman and Miller 2007).

A key result of this study was the reliable differentiation of trials requiring cue‐driven preference revision. Decoding performance revealed learning‐dependent shifts across regions: across learning days, OFC often showed stronger differentiation early in training, whereas ACC tended to exhibit stronger decoding once cue‐reward associations were stabilized. This pattern suggests that cue‐driven updates to relative EV may initially rely more heavily on value evaluation processes in OFC, while ACC may contribute increasingly to monitoring and updating evolving decision policies as learning progresses. Such distributed and dynamic contributions of prefrontal subregions during decision formation are consistent with prior work demonstrating temporally evolving choice signals across PFC circuits (Hunt et al. 2015; Kennerley and Wallis 2009). Control multivariate analyses confirmed that these revision‐related signals were not fully explained by overlapping cue variables, supporting a distributed implementation of preference updating across prefrontal circuits. Although we interpret these signals as reflecting cue‐driven preference revision, Hγ modulation during these trials may also reflect related processes such as decision conflict, salience, or response adjustment accompanying policy revision. Because our operational definition is based on changes in relative EV rather than latent belief states, the present analyses cannot dissociate cue‐driven preference revision from related processes such as decision conflict, salience, or response adjustment accompanying policy revision.

Prior to movement, Hγ activity in ACC, DLPFC, and OFC all contributed to the differentiation of target choice and expected reward, although the relative strength of these effects varied across regions and subjects. Single‐channel analyses revealed that Hγ activity ramped during the decision epoch and peaked immediately prior to or around movement onset, consistent with neural processes involved in deliberation and action selection preceding movement. Regional decoding performance also varied across subjects and training days, suggesting learning‐related changes in value‐related neural sensitivity.

Outcome‐related signals were evident during the reward period. Decoding of outcome–probability contrasts corresponding to positive and negative prediction‐error conditions revealed reliable differentiation across ACC, DLPFC, and OFC, with the relative contribution of region varying across subjects and prediction‐error conditions. Decoding of rewarded versus unrewarded outcomes in the no PE condition yielded the highest accuracies, indicating that frontal Hγ is sensitive to the presence or absence of reward and underscores the importance of carefully defining decoding contrasts to control for confounding sensory or task‐related factors.

Our findings are consistent with recent work showing heterogeneous modulation of value‐related signals across neural populations. For example, Muller et al. (2024) reported that individual neurons exhibit diverse sensitivities to value predictions and prediction‐error signals. Similarly, our results show heterogeneous Hγ modulation across electrodes for different outcome‐related contrasts, suggesting that distributed neural populations within PFC contribute complementary information about reward expectations, preference revision, and outcome evaluation. Decoding performance varied across sessions (see Table S2), likely reflecting differences in electrode coverage and the presence of highly informative channels, consistent with heterogeneous sampling of prefrontal populations.

Several limitations should be considered when interpreting these findings. First, the dataset includes recordings from two animals with variable electrode coverage, which limits the extent to which region‐specific conclusions can be generalized to the broader primate population. However, consistent patterns were observed across multiple sessions and in both animals, despite variability in decoding performance across sessions likely driven by differences in electrode coverage. Broader generalization will benefit from future studies with larger samples and more uniform regional coverage. Second, decoding analyses relied on predefined decoding contrasts, which may not capture more complex nonlinear interactions among decision variables (e.g., nonlinear integration of reward and spatial information). Future studies could combine Hγ with spiking or multiband decoding and apply advanced techniques such as neural population latent state modelling or recurrent decoding frameworks.

Overall, these findings position Hγ activity as a rich neural correlate of decision‐related computations in prefrontal circuits. Although certain task variables exhibited stronger modulation in particular regions, all three regions contributed to multiple aspects of cue evaluation, decision formation, and outcome processing. These overlapping patterns are consistent with distributed and mixed‐selective representations across PFC, while still revealing regional biases in the strength and timing of task‐related modulation. Together, these results highlight the flexibility of PFC Hγ signals and support their potential utility for tracking decision‐related neural dynamics in real time.

## Author Contributions

**Renée Johnston:** writing – review and editing, writing – original draft, methodology, software, conceptualization, formal analysis, investigation. **Chadwick Boulay:** writing – review and editing, conceptualization. **Laurence Hunt:** investigation, data curation, conceptualization, resources. **Steven W. Kennerley:** investigation, data curation, conceptualization, resources, writing – review and editing. **Adam J. Sachs:** writing – review and editing, supervision, conceptualization.

## Ethics Statement

All experimental procedures were approved by the UCL Local Ethical Procedures Committee and the UK Home Office and were carried out in accordance with the UK Animals (Scientific Procedures) Act (Hunt, Malalasekera, de Berker, et al. 2018).

## Conflicts of Interest

The authors declare no conflicts of interest.

## Supporting information


**Figure S1:** Session‐level choice bias for each animal.
**Figure S2:** Error rates as a function of the correct option across recording sessions.
**Figure S3:** Control analysis evaluating the effect of line‐noise notch filtering on Hγ decoding performance.
**Figure S4:** Region‐specific decoding of reward outcome and prediction error contrasts using Hγ activity.
**Table S1:** Pairwise post‐hoc comparisons of decoding accuracy across prefrontal regions.
**Table S2:** Robustness of decoding accuracy to session exclusion.
**Table S3:** Proportion of Bonferroni‐significant Hγ channels across prefrontal regions for the task variables shown in Figures 2e‐f.
**Table S4:** Region‐level summary of graded preference‐change regression analyses.
**Table S5:** Channel‐level regression results for graded preference‐change analyses.
**Table S6:** Regional summary of multivariate sensitivity to cue‐driven preference revision around Cue3.
**Table S7:** Channel‐level multivariate regression results for cue‐driven preference revision around Cue3.

## Data Availability

The data that support the findings of this study are openly available in CRCNS.org at https://crcns.org/data‐sets/pfc/pfc‐7.
