# Supplementary material for: Distinct High‐Gamma Signals in Primate Prefrontal Cortex Differentiate Cue Information, Preference Revision, and Expected Reward During Value‐Based Decisions
Source: Eur J Neurosci. 2026 Aug 12;64(3):e70641. doi: 10.1111/ejn.70641 (PMC13463214; doi:10.1111/ejn.70641)
Supplement: Supplementary file 1 [file EJN-64-0-s001.docx]

**Supplementary Information**

Supplementary Figure 1. Session-level choice bias for each animal.
Scatter plots show the bias in left vs right choices across all sessions for Miles (left) and Frank (right). Each point corresponds to one session, with correct trials shown in blue and incorrect trials in red. A dashed horizontal line at zero indicates no bias (equal left and right choices). Session IDs are displayed along the x-axis.

Supplementary Figure 2. Error rates as a function of the correct option across recording sessions.
Error rates (number of incorrect trials divided by total trials) are shown separately for trials in which the left option was correct (blue) and right option was correct (red). Data are plotted for Miles (left) and Frank (right). Miles exhibited relatively balanced error rates across left and right options. Frank showed consistently higher error rates when the left option was correct, consistent with a rightward choice bias.

Supplementary Figure 3. Control analysis evaluating the effect of line-noise notch filtering on Hγ decoding performance.

(a) Cue-related variables analyzed in the study.

(b) Replication of the statistical modulation analysis shown in Fig. 2b, restricted to the Cue3 epoch. The number of channels exhibiting significant Hγ modulation for each task variable is shown for subject Miles (top row) and Frank (bottom row). Significance was assessed using linear regression models applied independently to each recording channel. Results are shown using a 50 Hz notch filter (left) and after applying additional harmonic notch filters at 100 Hz and 150 Hz (right). The distribution of significant channels across task variables remains highly similar across filtering approaches.

(c) Comparison of SVM decoding accuracy for Cue Value Level around Cue3 when using a 50 Hz notch filter alone vs. additional harmonic notch filters at 100 Hz and 150 Hz. Decoding was performed independently for each recording channel, and the maximum decoding accuracy within the −300 to 300 ms Cue3 epoch was computed per channel. Regional values (ACC, DLPFC, OFC) represent the mean decoding accuracy across channels. Differences between filtering approaches were minimal (<1% across regions and animals), indicating that harmonic line noise does not meaningfully influence decoding performance.

Supplementary Figure 4. Region-specific decoding of reward outcome and prediction error contrasts using Hγ activity.

Time-resolved SVM decoding accuracy for No Prediction Error (No PE; a,d), Positive Prediction Error (Positive PE; b,e), and Negative Prediction Error (Negative PE; c,f) contrasts, shown separately for ACC (green), DLPFC (brown), and OFC (teal) in subject Miles (a–c) and subject Frank (d–f). Decoding was performed using pooled pseudo-population datasets constructed from all recorded Hγ channels within each prefrontal region during Day 3 sessions. Colored traces indicate observed decoding accuracy, whereas darker traces indicate decoding performance obtained from shuffled labels (chance-level control). The gray shaded region denotes the 500-ms reward window used for outcome decoding analyses. These region-specific decoding results complement the pooled all-PFC decoding examples shown in Figure 8a–f and provide a temporal visualization of the regional decoding differences summarized quantitatively in Supplementary Table 1.

Supplementary Table 1. Pairwise post-hoc comparisons of decoding accuracy across prefrontal regions.
Decoding accuracy for different task variables and events in two participants (Frank and Miles) was estimated across 20 resampled trial subsets drawn from Day 3 sessions. Within each subset, SVM decoding accuracy was estimated using 5-fold cross-validation, repeated 20 times, and the reported values reflect the mean accuracy across repetitions. Regional differences were assessed using Kruskal–Wallis tests followed by Dunn–Šidák–adjusted post-hoc comparisons. The table lists mean SVM classification accuracy for each region, along with adjusted p-values, Cohen’s d effect sizes, and rank-biserial correlations.

| Subject | Variable | Time (ms) | Event | Group1 (Mean SVM Accuracy) | Group2 (Mean SVM Accuracy) | Dunn-Šidák  adjusted pValue | Cohen’s d | Rank-biserial correlation |
| --- | --- | --- | --- | --- | --- | --- | --- | --- |
| Frank | Cue Level | 100 | after Cue1 | ACC - 63.22% | **OFC - 72.58%** | 1.30E-03 | -3.03 | -0.97 |
| Frank | Cue Level | 100 | after Cue1 | **ACC - 63.22%** | DLPFC - 50.28% | 7.11E-04 | 4.19 | 1.00 |
| Frank | Cue Level | 100 | after Cue1 | **OFC - 72.58%** | DLPFC - 50.28% | 1.96E-12 | 8.27 | 1.00 |
| Miles | Cue Level | 100 | after Cue1 | ACC - 72.60% | **OFC - 86.96%** | 2.46E-04 | -4.72 | -1.00 |
| Miles | Cue Level | 100 | after Cue1 | **ACC - 72.60%** | DLPFC - 67.51% | 8.40E-03 | 1.80 | 0.83 |
| Miles | Cue Level | 100 | after Cue1 | **OFC - 86.96%** | DLPFC - 67.51% | 1.30E-11 | 6.47 | 1.00 |
| Frank | Cue Level | 100 | after Cue3 | ACC - 49.51% | **OFC - 68.45%** | 1.58E-09 | -3.86 | -1.00 |
| Frank | Cue Level | 100 | after Cue3 | ACC - 49.51% | DLPFC - 54.84% | 1.79E-01 | -1.07 | -0.49 |
| Frank | Cue Level | 100 | after Cue3 | **OFC - 68.45%** | DLPFC - 54.84% | 3.99E-05 | 3.03 | 0.95 |
| Miles | Cue Level | 100 | after Cue3 | ACC - 67.83% | **OFC - 81.73%** | 1.90E-11 | -5.16 | -1.00 |
| Miles | Cue Level | 100 | after Cue3 | ACC - 67.83% | **DLPFC - 72.90%** | 5.86E-03 | -1.92 | -0.84 |
| Miles | Cue Level | 100 | after Cue3 | **OFC - 81.73%** | DLPFC - 72.90% | 4.79E-04 | 3.13 | 0.96 |
| Frank | Cue Position | 50 | after Cue1 | ACC - 49.75% | **OFC - 54.30%** | 2.00E-03 | -2.40 | -0.94 |
| Frank | Cue Position | 50 | after Cue1 | ACC - 49.75% | **DLPFC - 65.60%** | 2.91E-12 | -8.34 | -1.00 |
| Frank | Cue Position | 50 | after Cue1 | OFC - 54.30% | **DLPFC - 65.60%** | 5.74E-04 | -5.54 | -1.00 |
| Miles | Cue Position | 50 | after Cue1 | ACC - 52.11% | OFC - 55.46% | 2.64E-02 | -1.51 | -0.72 |
| Miles | Cue Position | 50 | after Cue1 | ACC - 52.11% | **DLPFC - 73.48%** | 4.72E-11 | -9.55 | -1.00 |
| Miles | Cue Position | 50 | after Cue1 | OFC - 55.46% | **DLPFC - 73.48%** | 1.12E-04 | -11.23 | -1.00 |
| Frank | Cue Position | 100 | before Cue3 | **ACC - 60.80%** | OFC - 52.94% | 1.14E-03 | 2.62 | 0.95 |
| Frank | Cue Position | 100 | before Cue3 | ACC - 60.80% | **DLPFC - 66.66%** | 1.47E-03 | -2.66 | -0.94 |
| Frank | Cue Position | 100 | before Cue3 | OFC - 52.94% | **DLPFC - 66.66%** | 5.79E-12 | -5.14 | -1.00 |
| Miles | Cue Position | 100 | before Cue3 | ACC - 52.54% | **OFC - 57.76%** | 1.24E-03 | -3.30 | -0.98 |
| Miles | Cue Position | 100 | before Cue3 | ACC - 52.54% | **DLPFC - 72.13%** | 1.83E-12 | -10.08 | -1.00 |
| Miles | Cue Position | 100 | before Cue3 | OFC - 57.76% | **DLPFC - 72.13%** | 7.36E-04 | -8.14 | -1.00 |
| Frank | Pref. Revision (L) | 50 | after Cue3 | **ACC - 64.39%** | OFC - 53.35% | 9.73E-05 | 1.64 | 0.76 |
| Frank | Pref. Revision (L) | 50 | after Cue3 | **ACC - 64.39%** | DLPFC - 56.10% | 2.50E-03 | 1.16 | 0.63 |
| Frank | Pref. Revision (L) | 50 | after Cue3 | OFC - 53.35% | DLPFC - 56.10% | 8.00E-01 | -0.40 | -0.16 |
| Miles | Pref. Revision (L) | 50 | after Cue3 | **ACC - 73.04%** | OFC - 65.32% | 1.60E-03 | 1.47 | 0.77 |
| Miles | Pref. Revision (L) | 50 | after Cue3 | **ACC - 73.04%** | DLPFC - 61.78% | 3.74E-06 | 1.67 | 0.77 |
| Miles | Pref. Revision (L) | 50 | after Cue3 | OFC - 65.32% | DLPFC - 61.78% | 4.20E-01 | 0.61 | 0.38 |
| Frank | Pref. Revision (R) | 50 | after Cue3 | **ACC - 57.33%** | OFC - 53.27% | 6.04E-01 | 0.53 | 0.24 |
| Frank | Pref. Revision (R) | 50 | after Cue3 | **ACC - 57.33%** | DLPFC - 42.35% | 2.15E-06 | 1.89 | 0.88 |
| Frank | Pref. Revision (R) | 50 | after Cue3 | OFC - 53.27% | DLPFC - 42.35% | 3.64E-04 | 1.57 | 0.74 |
| Miles | Pref. Revision (R) | 50 | after Cue3 | ACC - 61.03% | **OFC - 66.71%** | 1.22E-03 | -1.15 | -0.62 |
| Miles | Pref. Revision (R) | 50 | after Cue3 | ACC - 61.03% | DLPFC - 63.79% | 1.68E-01 | -0.59 | -0.38 |
| Miles | Pref. Revision (R) | 50 | after Cue3 | OFC - 66.71% | DLPFC - 63.79% | 2.67E-01 | 0.63 | 0.33 |
| Frank | Pref. Revision (L+R) | 50 | after Cue3 | ACC - 48.24% | OFC - 53.69% | 2.41E-02 | -0.82 | -0.46 |
| Frank | Pref. Revision (L+R) | 50 | after Cue3 | ACC - 48.24% | DLPFC - 51.60% | 3.22E-01 | -0.61 | -0.32 |
| Frank | Pref. Revision (L+R) | 50 | after Cue3 | OFC - 53.69% | DLPFC - 51.60% | 6.13E-01 | 0.34 | 0.24 |
| Miles | Pref. Revision (L+R) | 50 | after Cue3 | ACC - 69.93% | OFC - 71.75% | 4.03E-01 | -0.59 | -0.29 |
| Miles | Pref. Revision (L+R) | 50 | after Cue3 | **ACC - 69.93%** | DLPFC - 66.06% | 3.18E-03 | 1.26 | 0.63 |
| Miles | Pref. Revision (L+R) | 50 | after Cue3 | **OFC - 71.75%** | DLPFC - 66.06% | 8.35E-06 | 1.74 | 0.84 |
| Frank | Target Choice | 500 | before Move | **ACC - 57.03%** | OFC - 53.36% | 1.06E-03 | 1.41 | 0.69 |
| Frank | Target Choice | 500 | before Move | **ACC - 57.03%** | DLPFC - 51.85% | 5.92E-07 | 2.49 | 0.93 |
| Frank | Target Choice | 500 | before Move | OFC - 53.36% | DLPFC - 51.85% | 2.79E-01 | 0.53 | 0.33 |
| Miles | Target Choice | 500 | before Move | **ACC - 65.98%** | OFC - 50.96% | 1.77E-12 | 6.82 | 1.00 |
| Miles | Target Choice | 500 | before Move | **ACC - 65.98%** | DLPFC - 60.08% | 1.08E-03 | 2.73 | 0.98 |
| Miles | Target Choice | 500 | before Move | OFC - 50.96% | **DLPFC - 60.08%** | 8.33E-04 | -3.83 | -1.00 |
| Frank | Expected Reward | 250 | after Move | **ACC - 65.45%** | OFC - 58.59% | 4.27E-06 | 2.17 | 0.88 |
| Frank | Expected Reward | 250 | after Move | ACC - 65.45% | DLPFC - 65.42% | 1.00E+00 | 0.01 | 0.03 |
| Frank | Expected Reward | 250 | after Move | OFC - 58.59% | **DLPFC - 65.42%** | 6.85E-06 | -2.19 | -0.88 |
| Miles | Expected Reward | 250 | after Move | **ACC - 83.77%** | OFC - 73.85% | 8.78E-04 | 5.66 | 1.00 |
| Miles | Expected Reward | 250 | after Move | **ACC - 83.77%** | DLPFC - 62.08% | 1.31E-12 | 11.26 | 1.00 |
| Miles | Expected Reward | 250 | after Move | **OFC - 73.85%** | DLPFC - 62.08% | 8.78E-04 | 5.71 | 1.00 |
| Frank | No PE | 100 | after Reward | ACC - 59.90% | **OFC - 79.26%** | 1.11E-09 | -7.68 | -1.00 |
| Frank | No PE | 100 | after Reward | ACC - 59.90% | **DLPFC - 76.53%** | 1.27E-05 | -5.91 | -1.00 |
| Frank | No PE | 100 | after Reward | OFC - 79.26% | DLPFC - 76.53% | 2.61E-01 | 0.89 | 0.46 |
| Miles | No PE | 100 | after Reward | **ACC - 90.62%** | OFC - 85.12% | 7.90E-04 | 3.24 | 0.99 |
| Miles | No PE | 100 | after Reward | **ACC - 90.62%** | DLPFC - 80.21% | 2.64E-12 | 6.87 | 1.00 |
| Miles | No PE | 100 | after Reward | **OFC - 85.12%** | DLPFC - 80.21% | 1.40E-03 | 2.80 | 0.96 |
| Frank | No PE | 300 | after Reward | ACC - 67.98% | **OFC - 87.02%** | 1.88E-10 | -6.56 | -1.00 |
| Frank | No PE | 300 | after Reward | ACC - 67.98% | DLPFC - 72.33% | 7.93E-02 | -1.23 | -0.61 |
| Frank | No PE | 300 | after Reward | **OFC - 87.02%** | DLPFC - 72.33% | 4.52E-05 | 4.65 | 1.00 |
| Miles | No PE | 300 | after Reward | **ACC - 94.86%** | OFC - 74.28% | 1.83E-12 | 12.14 | 1.00 |
| Miles | No PE | 300 | after Reward | **ACC - 94.86%** | DLPFC - 90.28% | 1.24E-03 | 3.02 | 0.98 |
| Miles | No PE | 300 | after Reward | OFC - 74.28% | **DLPFC - 90.28%** | 7.36E-04 | -9.39 | -1.00 |
| Frank | Positive PE | 300 | after Reward | ACC - 51.52% | **OFC - 61.37%** | 3.32E-08 | -3.11 | -0.95 |
| Frank | Positive PE | 300 | after Reward | ACC - 51.52% | DLPFC - 54.17% | 2.63E-01 | -0.67 | -0.41 |
| Frank | Positive PE | 300 | after Reward | **OFC - 61.37%** | DLPFC - 54.17% | 1.52E-04 | 2.06 | 0.85 |
| Miles | Positive PE | 300 | after Reward | ACC - 59.88% | OFC - 64.70% | 1.21E-02 | -1.29 | -0.61 |
| Miles | Positive PE | 300 | after Reward | ACC - 59.88% | DLPFC - 56.52% | 1.89E-01 | 0.76 | 0.41 |
| Miles | Positive PE | 300 | after Reward | **OFC - 64.70%** | DLPFC - 56.52% | 7.66E-06 | 1.89 | 0.79 |
| Frank | Negative PE | 300 | after Reward | ACC - 52.40% | OFC - 50.34% | 8.28E-01 | 0.28 | 0.27 |
| Frank | Negative PE | 300 | after Reward | ACC - 52.40% | **DLPFC - 62.72%** | 9.53E-05 | -2.36 | -0.90 |
| Frank | Negative PE | 300 | after Reward | OFC - 50.34% | **DLPFC - 62.72%** | 2.53E-06 | -1.75 | -0.78 |
| Miles | Negative PE | 300 | after Reward | ACC - 55.19% | **OFC - 65.74%** | 1.79E-05 | -2.34 | -0.89 |
| Miles | Negative PE | 300 | after Reward | ACC - 55.19% | DLPFC - 59.58% | 1.84E-01 | -0.63 | -0.28 |
| Miles | Negative PE | 300 | after Reward | OFC - 65.74% | DLPFC - 59.58% | 2.16E-02 | 0.93 | 0.44 |

Supplementary Table 2. Robustness of decoding accuracy to session exclusion.
Decoding performance (SVM accuracy, %) for each task variable and prefrontal subregion (ACC, DLPFC, OFC) is reported for both subjects (Miles: 8 sessions; Frank: 9 sessions). For each condition, decoding was performed using a pseudo-population approach pooling channels across sessions and evaluated using five-fold cross-validation. To assess sensitivity to session composition, a leave-one-session-out procedure was applied. For each exclusion, decoding accuracy was recomputed, and the minimum and maximum accuracies observed across excluded sessions are reported (“Min” and “Max”).

Because trial counts were balanced through random subsampling, the entire decoding procedure was repeated 20 times, and values are reported as mean ± standard deviation across repetitions. The reported ranges (Min–Max) reflect variability due to session exclusion, while the ± values reflect variability due to trial resampling.

| Subject | Variables | ACC  SVM (%) | | DLPFC  SVM (%) | | OFC  SVM (%) | |
| --- | --- | --- | --- | --- | --- | --- | --- |
|  |  | **Min** | **Max** | **Min** | **Max** | **Min** | **Max** |
| Miles  8 sessions | Cue Value Level | 69.64 ± 1.72 | 77.39 ± 2.24 | 67.70 ± 2.23 | 74.73 ± 2.32 | 70.43 ± 2.74 | 83.22 ± 1.99 |
|  | Cue Position | 53.56 ± 1.45 | 56.64 ± 1.52 | 70.39 ± 1.93 | 76.14 ± 1.92 | 56.94 ± 1.97 | 63.66 ± 2.20 |
|  | Cue-Driven Pref Rev | 73.43 ± 4.84 | 88.09 ± 4.43 | 60.16 ± 4.54 | 67.96 ± 3.07 | 68.07 ± 3.82 | 76.46 ± 4.22 |
|  | Expected Reward | 63.07 ± 1.75 | 70.16 ± 2.11 | 54.73 ± 2.06 | 58.71 ± 1.69 | 60.18 ± 2.11 | 65.64 ± 1.44 |
|  | Target Choice | 61.49 ± 1.19 | 66.05 ± 1.30 | 55.70 ± 1.89 | 59.72 ± 2.51 | 50.30 ± 1.74 | 54.09 ± 1.70 |
|  | No PE | 94.88 ± 0.93 | 97.47 ± 0.94 | 85.38 ± 1.65 | 93.05 ± 1.40 | 81.38 ± 2.04 | 86.59 ± 1.73 |
|  | Negative PE | 66.32 ± 2.31 | 76.77 ± 5.27 | 63.49 ± 3.83 | 70.62 ± 3.58 | 69.91 ± 3.81 | 78.36 ± 4.40 |
|  | Positive PE | 66.32 ± 2.31 | 71.84 ± 2.26 | 60.59 ± 2.41 | 64.89 ± 2.63 | 64.10 ± 2.68 | 73.89 ± 2.13 |
| Frank  9 sessions | Cue Value Level | 56.98 ± 2.28 | 63.41 ± 3.02 | 57.09 ± 3.40 | 65.43 ± 2.50 | 63.53 ± 2.84 | 72.95 ± 2.19 |
|  | Cue Position | 56.44 ± 2.09 | 64.69 ± 1.63 | 58.59 ± 3.13 | 67.46 ± 2.50 | 50.78 ± 1.82 | 55.33 ± 2.37 |
|  | Cue-Driven Pref Rev | 61.84 ± 3.52 | 73.84 ± 4.78 | 58.89 ± 3.74 | 70.85 ± 6.20 | 63.38 ± 4.35 | 77.03 ± 6.35 |
|  | Expected Reward | 58.34 ± 2.38 | 63.91 ± 1.81 | 53.31 ± 2.91 | 59.85 ± 1.78 | 56.42 ± 2.22 | 62.00 ± 2.32 |
|  | Target Choice | 56.09 ± 2.36 | 61.72 ± 2.03 | 52.19 ± 1.99 | 55.08 ± 1.92 | 54.62 ± 1.96 | 58.22 ± 2.59 |
|  | No PE | 74.38 ± 2.74 | 83.77 ± 1.23 | 72.03 ± 2.28 | 78.76 ± 2.06 | 84.93 ± 1.83 | 89.33 ± 1.97 |
|  | Negative PE | 62.65 ± 3.64 | 69.89 ± 4.27 | 59.84 ± 2.55 | 68.19 ± 4.02 | 57.08 ± 4.02 | 64.15 ± 4.21 |
|  | Positive PE | 57.39 ± 1.72 | 61.58 ± 2.86 | 57.67 ± 1.85 | 62.31 ± 2.58 | 63.33 ± 2.12 | 67.58 ± 2.09 |

Supplementary Table 3. Proportion of Bonferroni-significant Hγ channels across prefrontal regions for the task variables shown in Figures 2e–f. Values indicate the percentage of recorded channels within each region (ACC, DLPFC, OFC) that exhibited significant Hγ modulation for each task variable in subjects Miles and Frank. Exact channel counts (significant channels/total recorded channels) are also provided to facilitate comparisons across regions with unequal electrode sampling. These values complement the raw channel-count histograms shown in Figures 2e–f and allow assessment of regional modulation independent of differences in electrode coverage.

| **Task Variable** | **ACC**  **Miles Frank** | | **DLPFC**  **Miles Frank** | | **OFC**  **Miles Frank** | |
| --- | --- | --- | --- | --- | --- | --- |
| Cue Level  Cue 1 | 44.4%  (12/27) | 70.8%  (17/24) | 53.8%  (7/13) | 50.0%  (10/20) | 61.1%  (11/18) | 53.3%  (8/15) |
| Cue Level  Cue 2 | 29.6%  (8/27) | 45.8%  (11/24) | 61.5%  (8/13) | 30.0%  (6/20) | 55.6%  (10/18) | 60.0%  (9/15) |
| Cue Level  Cue 3 | 29.6% (8/27) | 29.2% (7/24) | 76.9% (10/13) | 35.0% (7/20) | 55.6% (10/18) | 66.7% (10/15) |
| Cue Level  Cue 4 | 18.5%  (5/27) | 16.7%  (4/24) | 30.8%  (4/13) | 10.0%  (2/20) | 38.9%  (7/18) | 13.3%  (2/15) |
| Cue Position  Cue 1 | 11.1%  (3/27) | 20.8%  (5/24) | 76.9%  (10/13) | 65.0%  (13/20) | 27.8%  (5/18) | 26.7%  (4/15) |
| Cue Position  Cue 2 | 14.8%  (4/27) | 16.7%  (4/24) | 69.2%  (9/13) | 45.0%  (9/20) | 33.3%  (6/18) | 20.0%  (3/15) |
| Cue Position  Cue 3 | 7.4% (2/27) | 25.0% (6/24) | 76.9% (10/13) | 50.0% (10/20) | 16.7% (3/18) | 13.3% (2/15) |
| Cue Position  Cue 4 | 11.1%  (3/27) | 4.2%  (1/24) | 23.1%  (3/13) | 5.0%  (1/20) | 0.0%  (0/18) | 0.0%  (0/15) |
| Preference Revision  Cue 2 | 33.3%  (9/27) | 41.7%  (10/24) | 61.5%  (8/13) | 10.0%  (2/20) | 55.6%  (10/18) | 46.7%  (7/15) |
| Preference Revision  Cue 3 | 40.7% (11/27) | 25.0% (6/24) | 53.8% (7/13) | 25.0% (5/20) | 50.0% (9/18) | 26.7% (4/15) |
| Preference Revision  Cue 4 | 33.3%  (9/27) | 16.7%  (4/24) | 30.8%  (4/13) | 5.0%  (1/20) | 16.7%  (3/18) | 0.0%  (0/15) |
| Expected Reward | 44.4% (12/27) | 29.2% (7/24) | 46.2% (6/13) | 15.0% (3/20) | 33.3% (6/18) | 26.7% (4/15) |
| Target Choice | 22.2% (6/27) | 25.0% (6/24) | 23.1% (3/13) | 20.0% (4/20) | 5.6% (1/18) | 40.0% (6/15) |
| Negative PE | 74.1% (20/27) | 8.3% (2/24) | 30.8% (4/13) | 10.0% (2/20) | 66.7% (12/18) | 20.0% (3/15) |
| Positive PE | 48.1% (13/27) | 25.0% (6/24) | 46.2% (6/13) | 20.0% (4/20) | 55.6% (10/18) | 26.7% (4/15) |
| No PE | 88.9% (24/27) | 45.8% (11/24) | 69.2% (9/13) | 50.0% (10/20) | 77.8% (14/18) | 60.0% (9/15) |

Supplementary Table 4. Region-level summary of graded preference-change regression analyses.
For each subject and PFC subregion, the table reports the number of channels included (Region (#Channels)), the percentage of channels showing significant relationships between Hγ power and signed preference change (ΔPref) or magnitude of preference change (|ΔPref|) using uncorrected p-values (% Sig), and the percentage surviving Benjamini–Hochberg false discovery rate correction (% Sig (FDR), α = 0.05). Median partial R² values are reported to summarize effect sizes across channels within each region. FDR correction was applied separately within each subject × region × regression.

|  | Region (#Channels) | % Sig  ΔPref | % Sig  \| ΔPref \| | % Sig (FDR)  ΔPref | % Sig (FDR)  \| ΔPref \| | Median partial R²  ΔPref | Median partial R²  \| ΔPref \| |
| --- | --- | --- | --- | --- | --- | --- | --- |
| MILES | ACC (27) | 18.5 | 11.1 | 3.7 | 0 | 0.0017 | 0.0009 |
|  | DLPFC (13) | 15.4 | 7.7 | 0 | 0 | 0.0028 | 0.0033 |
|  | OFC (18) | 0 | 27.8 | 0 | 16.7 | 0.0019 | 0.0023 |
| FRANK | ACC (24) | 8.3 | 20.8 | 0 | 12.5 | 0.0050 | 0.0037 |
|  | DLPFC (20) | 10 | 25.0 | 5.0 | 5.0 | 0.0027 | 0.0041 |
|  | OFC (15) | 20.0 | 33.3 | 0 | 13.3 | 0.0031 | 0.0059 |

Supplementary Table 5. Channel-level regression results for graded preference-change analyses.
For each recording channel, the table reports the subject, session identifier (SID), cortical region, and channel number, along with regression statistics relating trial-by-trial Hγ power to signed preference change (ΔPref) and magnitude of preference change (|ΔPref|) measured in the 50–550 ms window following Cue3 onset. For each regression, uncorrected p-values (pvalue), false discovery rate–corrected p-values (pvalue (FDR), Benjamini–Hochberg, α = 0.05), and partial R² values are provided. FDR correction was performed across channels within each subject × region × regression. Partial R² reflects the proportion of variance in Hγ power uniquely explained by the corresponding predictor. Channels surviving FDR correction (pvalue (FDR) < 0.05) are indicated by an asterisk (*).

| **Subject** | **SID** | **Region** | **Chan** | **pvalue**  **ΔPref** | **pvalue (FDR)**  **ΔPref** | **Partial R² ΔPref** | **pvalue**  **\| ΔPref \|** | **pvalue (FDR)**  **\|ΔPref\|** | **Partial R² \|ΔPref\|** |
| --- | --- | --- | --- | --- | --- | --- | --- | --- | --- |
| Miles | 7 | ACC | 20 | 0.4072 | 0.7482 | 0.0018 | 0.9151 | 0.9503 | 2.9e-05 |
|  | **7** | **ACC** | **23** | **5.2e-04** | **0.0141*** | **0.0303** | **0.6613** | **0.8116** | **4.9e-04** |
|  | 12 | ACC | 2 | 0.0047 | 0.0592 | 0.0204 | 0.0086 | 0.2241 | 0.0176 |
|  | 12 | ACC | 3 | 0.2646 | 0.6495 | 0.0032 | 0.0314 | 0.2823 | 0.0119 |
|  | 16 | ACC | 1 | 0.9551 | 0.9551 | 7.1e-06 | 0.6270 | 0.8062 | 5.3e-04 |
|  | 16 | ACC | 3 | 0.5588 | 0.7592 | 7.6e-04 | 0.1063 | 0.3686 | 0.0058 |
|  | 16 | ACC | 4 | 0.1418 | 0.4785 | 0.0048 | 0.4175 | 0.8062 | 0.0015 |
|  | 16 | ACC | 20 | 0.8496 | 0.9551 | 8.0e-05 | 0.0863 | 0.3686 | 0.0066 |
|  | 16 | ACC | 22 | 0.9347 | 0.9551 | 1.5e-05 | 0.6073 | 0.8062 | 5.9e-04 |
|  | 23 | ACC | 1 | 0.4647 | 0.7482 | 0.0012 | 0.5688 | 0.8062 | 7.1e-04 |
|  | 23 | ACC | 2 | 0.3290 | 0.7404 | 0.0021 | 0.6178 | 0.8062 | 5.4e-04 |
|  | 23 | ACC | 17 | 0.4672 | 0.7482 | 0.0012 | 0.8271 | 0.8933 | 1.0e-04 |
|  | 23 | ACC | 18 | 0.4711 | 0.7482 | 0.0011 | 0.5608 | 0.8062 | 7.4e-04 |
|  | 27 | ACC | 1 | 0.5304 | 0.7592 | 0.0012 | 0.1092 | 0.3686 | 0.0079 |
|  | 27 | ACC | 2 | 0.0088 | 0.0592 | 0.0211 | 0.5328 | 0.8062 | 0.0012 |
|  | 27 | ACC | 17 | 0.1920 | 0.5192 | 0.0053 | 0.5943 | 0.8062 | 8.8e-04 |
|  | 27 | ACC | 18 | 0.1320 | 0.4785 | 0.0070 | 0.6165 | 0.8062 | 7.8e-04 |
|  | 30 | ACC | 1 | 0.1324 | 0.4785 | 0.0053 | 0.0166 | 0.2241 | 0.0133 |
|  | 30 | ACC | 3 | 0.9092 | 0.9551 | 3.0e-05 | 0.7975 | 0.8933 | 1.5e-04 |
|  | 30 | ACC | 17 | 0.9024 | 0.9551 | 3.5e-05 | 0.4983 | 0.8062 | 0.0011 |
|  | 30 | ACC | 18 | 0.0192 | 0.1039 | 0.0127 | 0.0925 | 0.3686 | 0.0066 |
|  | 30 | ACC | 19 | 0.1923 | 0.5192 | 0.0040 | 0.3671 | 0.8062 | 0.0019 |
|  | 42 | ACC | 23 | 0.7466 | 0.9457 | 2.5e-04 | 0.9901 | 0.9901 | 3.6e-07 |
|  | 46 | ACC | 19 | 0.5624 | 0.7592 | 7.6e-04 | 0.0651 | 0.3686 | 0.0076 |
|  | 46 | ACC | 20 | 0.7706 | 0.9457 | 1.9e-04 | 0.2986 | 0.8062 | 0.0024 |
|  | 46 | ACC | 22 | 0.0079 | 0.0592 | 0.0158 | 0.7580 | 0.8899 | 2.1e-04 |
|  | 46 | ACC | 24 | 0.3795 | 0.7482 | 0.0017 | 0.6211 | 0.8062 | 5.5e-04 |
|  | 12 | DLPFC | 7 | 0.1160 | 0.3429 | 0.0063 | 0.7899 | 0.9335 | 1.8e-04 |
|  | 12 | DLPFC | 8 | 0.4195 | 0.4958 | 0.0017 | 0.1985 | 0.4162 | 0.0042 |
|  | 16 | DLPFC | 5 | 0.0710 | 0.3077 | 0.0073 | 0.4634 | 0.7530 | 0.0012 |
|  | 16 | DLPFC | 6 | 0.2623 | 0.4520 | 0.0028 | 0.7642 | 0.9335 | 2.0e-04 |
|  | 16 | DLPFC | 17 | 0.9818 | 0.9818 | 1.2e-06 | 0.9260 | 0.9806 | 1.9e-05 |
|  | 16 | DLPFC | 18 | 0.3632 | 0.4722 | 0.0018 | 0.2241 | 0.4162 | 0.0033 |
|  | 30 | DLPFC | 7 | 0.2781 | 0.4520 | 0.0027 | 0.7887 | 0.9335 | 1.7e-04 |
|  | 30 | DLPFC | 8 | 0.3273 | 0.4722 | 0.0022 | 0.9806 | 0.9806 | 1.4e-06 |
|  | 42 | DLPFC | 9 | 0.5526 | 0.5987 | 8.3e-04 | 0.1991 | 0.4162 | 0.0039 |
|  | 42 | DLPFC | 10 | 0.1319 | 0.3429 | 0.0053 | 0.1161 | 0.3772 | 0.0058 |
|  | 42 | DLPFC | 20 | 0.0156 | 0.1255 | 0.0137 | 0.0933 | 0.3772 | 0.0066 |
|  | 46 | DLPFC | 3 | 0.0193 | 0.1255 | 0.0123 | 0.0071 | 0.0922 | 0.0162 |
|  | 46 | DLPFC | 4 | 0.2008 | 0.4351 | 0.0037 | 0.0545 | 0.3542 | 0.0083 |
|  | 7 | OFC | 18 | 0.4107 | 0.5686 | 0.0017 | 0.8266 | 0.9798 | 1.2e-04 |
|  | 23 | OFC | 4 | 0.7565 | 0.8009 | 2.1e-04 | 0.5306 | 0.7452 | 8.6e-04 |
|  | 27 | OFC | 3 | 0.3635 | 0.5686 | 0.0026 | 0.9111 | 0.9798 | 3.9e-05 |
|  | 27 | OFC | 4 | 0.4067 | 0.5686 | 0.0021 | 0.9798 | 0.9798 | 2.0e-06 |
|  | 27 | OFC | 19 | 0.6654 | 0.7782 | 5.8e-04 | 0.0236 | 0.1063 | 0.0158 |
|  | 30 | OFC | 5 | 0.6406 | 0.7782 | 5.1e-04 | 0.0887 | 0.2660 | 0.0067 |
|  | 30 | OFC | 6 | 0.4062 | 0.5686 | 0.0016 | 0.4908 | 0.7452 | 0.0011 |
|  | 30 | OFC | 9 | 0.2082 | 0.5686 | 0.0037 | 0.5382 | 0.7452 | 8.8e-04 |
|  | 30 | OFC | 10 | 0.1072 | 0.5686 | 0.0060 | 0.8772 | 0.9798 | 5.6e-05 |
|  | 30 | OFC | 22 | 0.6917 | 0.7782 | 3.7e-04 | 0.2411 | 0.5424 | 0.0032 |
|  | 30 | OFC | 24 | 0.3882 | 0.5686 | 0.0017 | 0.9287 | 0.9798 | 1.9e-05 |
|  | 42 | OFC | 4 | 0.2533 | 0.5686 | 0.0031 | 0.3089 | 0.6015 | 0.0024 |
|  | **42** | **OFC** | **8** | **0.4004** | **0.5686** | **0.0017** | **5.9e-04** | **0.0053*** | **0.0274** |
|  | 42 | OFC | 11 | 0.1903 | 0.5686 | 0.0040 | 0.3342 | 0.6015 | 0.0022 |
|  | 46 | OFC | 1 | 0.2452 | 0.5686 | 0.0030 | 0.1667 | 0.4287 | 0.0043 |
|  | **46** | **OFC** | **2** | **0.2955** | **0.5686** | **0.0025** | **6.0e-08** | **1.1e-06*** | **0.0641** |
|  | **46** | **OFC** | **5** | **0.0706** | **0.5686** | **0.0073** | **0.0036** | **0.0215*** | **0.0190** |
|  | 46 | OFC | 6 | 0.9481 | 0.9481 | 9.5e-06 | 0.0491 | 0.1768 | 0.0087 |
| Frank | 4 | ACC | 1 | 0.0905 | 0.3619 | 0.0085 | 0.6599 | 0.8794 | 5.8e-04 |
|  | 4 | ACC | 2 | 0.3567 | 0.6116 | 0.0025 | 0.7006 | 0.8794 | 4.4e-04 |
|  | 4 | ACC | 3 | 0.0641 | 0.3619 | 0.0102 | 0.5524 | 0.8286 | 0.0011 |
|  | 4 | ACC | 4 | 0.6525 | 0.9441 | 6.1e-04 | 0.4853 | 0.7934 | 0.0015 |
|  | 4 | ACC | 17 | 0.9441 | 0.9441 | 1.5e-05 | 0.1518 | 0.3828 | 0.0061 |
|  | 4 | ACC | 18 | 0.7207 | 0.9441 | 3.8e-04 | 0.7586 | 0.8794 | 2.8e-04 |
|  | 4 | ACC | 19 | 0.9155 | 0.9441 | 3.4e-05 | 0.9618 | 0.9618 | 6.9e-06 |
|  | 4 | ACC | 20 | 0.8775 | 0.9441 | 7.1e-05 | 0.1595 | 0.3828 | 0.0059 |
|  | 10 | ACC | 1 | 0.1749 | 0.4197 | 0.0062 | 0.0500 | 0.2398 | 0.0129 |
|  | **10** | **ACC** | **2** | **0.0734** | **0.3619** | **0.0108** | **3.2e-04** | **0.0038*** | **0.0429** |
|  | 10 | ACC | 3 | 0.2899 | 0.5352 | 0.0038 | 0.3175 | 0.6351 | 0.0034 |
|  | 10 | ACC | 17 | 0.1361 | 0.3702 | 0.0075 | 0.0130 | 0.0777 | 0.0207 |
|  | 10 | ACC | 18 | 0.0145 | 0.3103 | 0.0200 | 6.9e-04 | 0.0056 | 0.0382 |
|  | 10 | ACC | 19 | 0.0259 | 0.3103 | 0.0167 | 0.8061 | 0.8794 | 2.0e-04 |
|  | 10 | ACC | 20 | 0.8025 | 0.9441 | 2.1e-04 | 0.1594 | 0.3828 | 0.0067 |
|  | 20 | ACC | 1 | 0.8299 | 0.9441 | 2.0e-04 | 0.1041 | 0.3569 | 0.0113 |
|  | 20 | ACC | 2 | 0.6698 | 0.9441 | 7.8e-04 | 0.1951 | 0.4257 | 0.0072 |
|  | 24 | ACC | 1 | 0.3916 | 0.6265 | 0.0048 | 0.4285 | 0.7910 | 0.0041 |
|  | 24 | ACC | 2 | 0.1188 | 0.3702 | 0.0157 | 0.0678 | 0.2714 | 0.0215 |
|  | 25 | ACC | 1 | 0.2077 | 0.4531 | 0.0088 | 0.4959 | 0.7934 | 0.0026 |
|  | 25 | ACC | 2 | 0.1388 | 0.3702 | 0.0121 | 0.7837 | 0.8794 | 4.2e-04 |
|  | 30 | ACC | 17 | 0.0844 | 0.3619 | 0.0095 | 0.7088 | 0.8794 | 4.4e-04 |
|  | 30 | ACC | 18 | 0.9012 | 0.9441 | 4.9e-05 | 0.8889 | 0.9276 | 6.2e-05 |
|  | **31** | **ACC** | **17** | **0.2390** | **0.4781** | **0.0053** | **4.0e-06** | **9.5e-05*** | **0.0787** |
|  | 4 | DLPFC | 8 | 0.4096 | 0.5998 | 0.0020 | 0.5189 | 0.5541 | 0.0012 |
|  | 4 | DLPFC | 21 | 0.4319 | 0.5998 | 0.0018 | 0.0308 | 0.1540 | 0.0138 |
|  | 4 | DLPFC | 22 | 0.0226 | 0.2258 | 0.0154 | 0.4501 | 0.5541 | 0.0017 |
|  | 4 | DLPFC | 23 | 0.0515 | 0.3433 | 0.0113 | 0.1948 | 0.5141 | 0.0050 |
|  | 4 | DLPFC | 24 | 0.5899 | 0.6939 | 8.7e-04 | 0.5264 | 0.5541 | 0.0012 |
|  | 10 | DLPFC | 22 | 0.2600 | 0.5998 | 0.0043 | 0.3232 | 0.5141 | 0.0033 |
|  | 10 | DLPFC | 23 | 0.8159 | 0.9066 | 1.8e-04 | 0.0883 | 0.2943 | 0.0098 |
|  | **10** | **DLPFC** | **24** | **0.0013** | **0.0266*** | **0.0343** | **0.3521** | **0.5141** | **0.0029** |
|  | 20 | DLPFC | 7 | 0.5065 | 0.6331 | 0.0019 | 0.3026 | 0.5141 | 0.0046 |
|  | 20 | DLPFC | 8 | 0.2051 | 0.5998 | 0.0069 | 0.3019 | 0.5141 | 0.0046 |
|  | 20 | DLPFC | 17 | 0.4409 | 0.5998 | 0.0026 | 0.6066 | 0.6066 | 0.0011 |
|  | 20 | DLPFC | 18 | 0.9231 | 0.9231 | 4.0e-05 | 0.0249 | 0.1540 | 0.0214 |
|  | 20 | DLPFC | 19 | 0.1578 | 0.5998 | 0.0085 | 0.4109 | 0.5479 | 0.0029 |
|  | 20 | DLPFC | 20 | 0.4110 | 0.5998 | 0.0029 | 0.3599 | 0.5141 | 0.0036 |
|  | 25 | DLPFC | 23 | 0.3629 | 0.5998 | 0.0046 | 0.4781 | 0.5541 | 0.0028 |
|  | 30 | DLPFC | 25 | 0.2473 | 0.5998 | 0.0043 | 0.2812 | 0.5141 | 0.0037 |
|  | 30 | DLPFC | 26 | 0.3772 | 0.5998 | 0.0025 | 0.2058 | 0.5141 | 0.0051 |
|  | 31 | DLPFC | 25 | 0.9026 | 0.9231 | 5.8e-05 | 7.3e-04 | 0.0147 | 0.0430 |
|  | 31 | DLPFC | 26 | 0.1928 | 0.5998 | 0.0065 | 0.0118 | 0.1179 | 0.0241 |
|  | 34 | DLPFC | 21 | 0.4498 | 0.5998 | 0.0021 | 0.0466 | 0.1865 | 0.0142 |
|  | 10 | OFC | 5 | 0.2512 | 0.5382 | 0.0044 | 0.0150 | 0.0749 | 0.0198 |
|  | **10** | **OFC** | **6** | **0.0143** | **0.1072** | **0.0201** | **0.0021** | **0.0159*** | **0.0314** |
|  | 10 | OFC | 7 | 0.0083 | 0.1072 | 0.0233 | 0.4722 | 0.6439 | 0.0017 |
|  | 10 | OFC | 8 | 0.7909 | 0.8910 | 2.4e-04 | 0.2044 | 0.3833 | 0.0054 |
|  | 24 | OFC | 17 | 0.8912 | 0.8912 | 1.2e-04 | 0.1584 | 0.3395 | 0.0129 |
|  | 24 | OFC | 18 | 0.7901 | 0.8910 | 4.6e-04 | 0.1304 | 0.3260 | 0.0148 |
|  | 25 | OFC | 7 | 0.0989 | 0.2578 | 0.0150 | 0.8435 | 0.9038 | 2.2e-04 |
|  | 25 | OFC | 8 | 0.0320 | 0.1598 | 0.0252 | 0.0289 | 0.1040 | 0.0261 |
|  | 25 | OFC | 21 | 0.0689 | 0.2578 | 0.0182 | 0.3018 | 0.4527 | 0.0059 |
|  | 25 | OFC | 22 | 0.6964 | 0.8910 | 8.4e-04 | 0.6160 | 0.7700 | 0.0014 |
|  | 30 | OFC | 27 | 0.1031 | 0.2578 | 0.0084 | 0.0347 | 0.1040 | 0.0141 |
|  | 30 | OFC | 28 | 0.4084 | 0.6806 | 0.0022 | 0.2991 | 0.4527 | 0.0034 |
|  | 31 | OFC | 27 | 0.3684 | 0.6806 | 0.0031 | 0.7592 | 0.8760 | 3.6e-04 |
|  | **31** | **OFC** | **28** | **0.8316** | **0.8910** | **1.7e-04** | **1.5e-04** | **0.0023*** | **0.0539** |
|  | 34 | OFC | 25 | 0.6517 | 0.8910 | 7.3e-04 | 0.9107 | 0.9107 | 4.5e-05 |

Supplementary Table 6. Regional summary of multivariate sensitivity to cue-driven preference revision around Cue3.

For each subject and region (ACC, DLPFC, OFC), we report the total number of recorded channels and the percentage showing a significant effect of cue-driven preference revision in a multivariate regression model. The model simultaneously included preference revision (update vs confirmation), cue level, and cue position as regressors. Significance was determined using the minimum p-value for the preference revision regressor across the −300 to +300 ms interval. Raw percentages reflect channels with p < 0.05. FDR-corrected percentages reflect channels surviving Benjamini–Hochberg correction (α = 0.05) applied within each subject and region.

|  | Region | #Channels | % Sig (Multivariate)  Cue-Driven Preference Revision | % Sig (FDR; Multivariate)  Cue-Driven Preference Revision |
| --- | --- | --- | --- | --- |
| Miles | ACC | 27 | 37.04 | 0 |
|  | DLPFC | 13 | 30.77 | 7.69 |
|  | OFC | 18 | 27.78 | 22.22 |
| Frank | ACC | 24 | 20.83 | 4.17 |
|  | DLPFC | 20 | 40.00 | 20.00 |
|  | OFC | 15 | 20.00 | 13.33 |

Supplementary Table 7. Channel-level multivariate regression results for cue-driven preference revision around Cue3.

For each channel, we fit a multivariate linear regression model to Hγ power in sliding windows around Cue3. The model included cue-driven preference revision (categorical: update vs confirmation), cue level, and cue position as simultaneous regressors. For each channel, the minimum p-value associated with the preference revision regressor across the −300 to +300 ms interval is reported (p_min). False discovery rate (FDR)–corrected p-values (q) were computed using the Benjamini–Hochberg procedure within each subject and region (ACC, DLPFC, OFC). Channels surviving FDR correction (pvalue (FDR) < 0.05) are indicated by an asterisk (*).

| **Subject** | **SID** | **Region** | **Chan** | **Minimum pvalue (Multivariate)**  **Cue-Driven Preference Revision** | **Minimum pvalue (FDR; Multivariate)**  **Cue-Driven Preference Revision** |
| --- | --- | --- | --- | --- | --- |
| Miles | 7 | ACC | 20 | 0.0287 | 0.1325 |
|  | 7 | ACC | 23 | 0.0577 | 0.1342 |
|  | 12 | ACC | 2 | 0.3888 | 0.4199 |
|  | 12 | ACC | 3 | 0.0748 | 0.1342 |
|  | 16 | ACC | 1 | 0.2918 | 0.3550 |
|  | 16 | ACC | 3 | 0.0393 | 0.1325 |
|  | 16 | ACC | 4 | 0.4354 | 0.4521 |
|  | 16 | ACC | 20 | 0.1710 | 0.2565 |
|  | 16 | ACC | 22 | 0.2091 | 0.2972 |
|  | 23 | ACC | 1 | 0.0795 | 0.1342 |
|  | 23 | ACC | 2 | 0.0744 | 0.1342 |
|  | 23 | ACC | 17 | 0.0280 | 0.1325 |
|  | 23 | ACC | 18 | 0.0676 | 0.1342 |
|  | 27 | ACC | 1 | 0.2370 | 0.3047 |
|  | 27 | ACC | 2 | 0.0189 | 0.1325 |
|  | 27 | ACC | 17 | 0.0313 | 0.1325 |
|  | 27 | ACC | 18 | 0.3570 | 0.4017 |
|  | 30 | ACC | 1 | 0.0458 | 0.1339 |
|  | 30 | ACC | 3 | 0.0291 | 0.1325 |
|  | 30 | ACC | 17 | 0.0973 | 0.1545 |
|  | 30 | ACC | 18 | 0.0496 | 0.1339 |
|  | 30 | ACC | 19 | 0.0371 | 0.1325 |
|  | 42 | ACC | 23 | 0.3024 | 0.3550 |
|  | 46 | ACC | 19 | 0.2351 | 0.3047 |
|  | 46 | ACC | 20 | 0.4799 | 0.4799 |
|  | 46 | ACC | 22 | 0.0640 | 0.1342 |
|  | 46 | ACC | 24 | 0.0171 | 0.1325 |
|  | 12 | DLPFC | 7 | 0.0539 | 0.1401 |
|  | 12 | DLPFC | 8 | 0.1236 | 0.2009 |
|  | 16 | DLPFC | 5 | 0.0309 | 0.1005 |
|  | 16 | DLPFC | 6 | 0.0267 | 0.1005 |
|  | 16 | DLPFC | 17 | 0.3238 | 0.3883 |
|  | 16 | DLPFC | 18 | 0.0083 | 0.0537 |
|  | 30 | DLPFC | 7 | 0.3584 | 0.3883 |
|  | **30** | **DLPFC** | **8** | **0.0010** | **0.0133*** |
|  | 42 | DLPFC | 9 | 0.1198 | 0.2009 |
|  | 42 | DLPFC | 10 | 0.6009 | 0.6009 |
|  | 42 | DLPFC | 20 | 0.0846 | 0.1833 |
|  | 46 | DLPFC | 3 | 0.3367 | 0.3883 |
|  | 46 | DLPFC | 4 | 0.1680 | 0.2426 |
|  | 7 | OFC | 18 | 0.0541 | 0.1284 |
|  | 23 | OFC | 4 | 0.0713 | 0.1284 |
|  | 27 | OFC | 3 | 0.1710 | 0.2198 |
|  | 27 | OFC | 4 | 0.2222 | 0.2499 |
|  | 27 | OFC | 19 | 0.0624 | 0.1284 |
|  | **30** | **OFC** | **5** | **0.0070** | **0.0430*** |
|  | **30** | **OFC** | **6** | **9.7847e-04** | **0.0176*** |
|  | 30 | OFC | 9 | 0.1392 | 0.2112 |
|  | 30 | OFC | 10 | 0.0658 | 0.1284 |
|  | 30 | OFC | 22 | 0.0235 | 0.0845 |
|  | **30** | **OFC** | **24** | **0.0096** | **0.0430*** |
|  | **42** | **OFC** | **4** | **0.0081** | **0.0430*** |
|  | 42 | OFC | 8 | 0.1513 | 0.2112 |
|  | 42 | OFC | 11 | 0.0519 | 0.1284 |
|  | 46 | OFC | 1 | 0.1525 | 0.2112 |
|  | 46 | OFC | 2 | 0.3985 | 0.4220 |
|  | 46 | OFC | 5 | 0.4742 | 0.4742 |
|  | 46 | OFC | 6 | 0.2093 | 0.2499 |
| Frank | 4 | ACC | 1 | 0.4687 | 0.4687 |
|  | 4 | ACC | 2 | 0.4142 | 0.4322 |
|  | 4 | ACC | 3 | 0.3867 | 0.4218 |
|  | 4 | ACC | 4 | 0.0727 | 0.2122 |
|  | 4 | ACC | 17 | 0.0796 | 0.2122 |
|  | 4 | ACC | 18 | 0.3385 | 0.3869 |
|  | 4 | ACC | 19 | 0.1191 | 0.2382 |
|  | 4 | ACC | 20 | 0.2436 | 0.3309 |
|  | 10 | ACC | 1 | 0.0330 | 0.1704 |
|  | 10 | ACC | 2 | 0.2497 | 0.3309 |
|  | 10 | ACC | 3 | 0.0291 | 0.1704 |
|  | 10 | ACC | 17 | 0.0629 | 0.2122 |
|  | 10 | ACC | 18 | 0.0698 | 0.2122 |
|  | 10 | ACC | 19 | 0.0165 | 0.1704 |
|  | 10 | ACC | 20 | 0.2662 | 0.3309 |
|  | 20 | ACC | 1 | 0.1047 | 0.2284 |
|  | 20 | ACC | 2 | 0.0355 | 0.1704 |
|  | 24 | ACC | 1 | 0.1700 | 0.2915 |
|  | 24 | ACC | 2 | 0.1531 | 0.2827 |
|  | 25 | ACC | 1 | 0.2164 | 0.3246 |
|  | 25 | ACC | 2 | 0.0897 | 0.2154 |
|  | 30 | ACC | 17 | 0.2757 | 0.3309 |
|  | **30** | **ACC** | **18** | **0.0018** | **0.0443*** |
|  | 31 | ACC | 17 | 0.1955 | 0.3129 |
|  | 4 | DLPFC | 8 | 0.0710 | 0.1520 |
|  | 4 | DLPFC | 21 | 0.2949 | 0.4213 |
|  | 4 | DLPFC | 22 | 0.0230 | 0.0664 |
|  | 4 | DLPFC | 23 | 0.1543 | 0.2806 |
|  | **4** | **DLPFC** | **24** | **3.5502e-04** | **0.0071*** |
|  | 10 | DLPFC | 22 | 0.3893 | 0.4326 |
|  | 10 | DLPFC | 23 | 0.5877 | 0.6009 |
|  | 10 | DLPFC | 24 | 0.6009 | 0.6009 |
|  | **20** | **DLPFC** | **7** | **0.0060** | **0.0298*** |
|  | 20 | DLPFC | 8 | 0.3623 | 0.4262 |
|  | 20 | DLPFC | 17 | 0.3167 | 0.4223 |
|  | 20 | DLPFC | 18 | 0.2455 | 0.3777 |
|  | 20 | DLPFC | 19 | 0.2005 | 0.3342 |
|  | 20 | DLPFC | 20 | 0.3467 | 0.4262 |
|  | **25** | **DLPFC** | **23** | **0.0043** | **0.0288*** |
|  | 30 | DLPFC | 25 | 0.0265 | 0.0664 |
|  | 30 | DLPFC | 26 | 0.0760 | 0.1520 |
|  | 31 | DLPFC | 25 | 0.0135 | 0.0539 |
|  | **31** | **DLPFC** | **26** | **0.0022** | **0.0222*** |
|  | 34 | DLPFC | 21 | 0.0254 | 0.0664 |
|  | 10 | OFC | 5 | 0.2773 | 0.3467 |
|  | 10 | OFC | 6 | 0.4293 | 0.4293 |
|  | 10 | OFC | 7 | 0.0818 | 0.2454 |
|  | **10** | **OFC** | **8** | **0.0030** | **0.0322*** |
|  | 24 | OFC | 17 | 0.1501 | 0.3075 |
|  | 24 | OFC | 18 | 0.3981 | 0.4293 |
|  | 25 | OFC | 7 | 0.0593 | 0.2224 |
|  | 25 | OFC | 8 | 0.2139 | 0.3075 |
|  | 25 | OFC | 21 | 0.2051 | 0.3075 |
|  | 25 | OFC | 22 | 0.2192 | 0.3075 |
|  | **30** | **OFC** | **27** | **0.0043** | **0.0322*** |
|  | 30 | OFC | 28 | 0.4070 | 0.4293 |
|  | 31 | OFC | 27 | 0.1409 | 0.3075 |
|  | 31 | OFC | 28 | 0.2255 | 0.3075 |
|  | 34 | OFC | 25 | 0.0430 | 0.2148 |
